# Laser-induced incandescence for non-soot nanoparticles: recent trends and current challenges

**DOI:** 10.1007/s00340-022-07769-z

**Published:** 2022-03-14

**Authors:** Timothy A. Sipkens, Jan Menser, Thomas Dreier, Christof Schulz, Gregory J. Smallwood, Kyle J. Daun

**Affiliations:** 1 Metrology Research Centre, National Research Council Canada, Ottawa, K1K 2E1 Canada; 2 IVG, Institute for Combustion and Gas Dynamics – Reactive Fluids, and CENIDE, Center for Nanointegration Duisburg Essen, University of Duisburg-Essen, 47057 Duisburg, Germany; 3 Department of Mechanical and Mechatronics Engineering, University of Waterloo, Waterloo, N2L 3G1 Canada

## Abstract

Laser-induced incandescence (LII) is a widely used combustion diagnostic for in situ measurements of soot primary particle sizes and volume fractions in flames, exhaust gases, and the atmosphere. Increasingly, however, it is applied to characterize engineered nanomaterials, driven by the increasing industrial relevance of these materials and the fundamental scientific insights that may be obtained from these measurements. This review describes the state of the art as well as open research challenges and new opportunities that arise from LII measurements on non-soot nanoparticles. An overview of the basic LII model, along with statistical techniques for inferring quantities-of-interest and associated uncertainties is provided, with a review of the application of LII to various classes of materials, including elemental particles, oxide and nitride materials, and non-soot carbonaceous materials, and core–shell particles. The paper concludes with a discussion of combined and complementary diagnostics, and an outlook of future research.

## Introduction

Gas-phase synthesis of nanoparticles offers the possibility of generating high-purity materials via a continuous flow process. Controlled variation of size and morphology at the nanoscale brings exciting new possibilities for the design of materials having unique size-dependent properties that enhance the performance of current devices and processes, and lays a foundation for emerging technology [1]. Applications include electronics, catalysis, batteries, photovoltaics, biological and biomedical applications, gas sensing, among others [2, 3]. The functionality of these materials depends strongly on the size and morphology of individual particles, and, in some cases, aggregates and agglomerates [4]. These attributes are controlled by varying reaction conditions, including precursor composition, gas temperature, and flow rate/reaction time [5, 6]. When designing and operating gas-phase nanoparticle synthesis processes, it is crucial to identify and control the formation conditions to produce materials having a narrow band of characteristics. Knowledge of particle size and morphology throughout the reactor is, therefore, needed to establish the operating conditions that lead to the production of nanoparticles having the desired characteristics, and also as a means for online process control.

While ex situ techniques are often considered the “gold standard” for characterizing the product properties, they do not possess the spatial resolution needed to map out nanoparticle attributes within the reactor flow field, nor do they have the temporal resolution needed for online control. Moreover, extracting particles from within a reactor is complicated by limited physical access [7], and the extraction process can introduce sampling biases that are difficult to characterize [8]. Therefore, optical in situ diagnostics are highly desired for gaining fundamental understanding of particle formation and growth in model experiments as well as in production reactors, and can also provide critical data for model validation in practical synthesis configurations [9].

Optical diagnostics have long been used to characterize soot particle sizes, morphologies, and volume fractions within flames and engines, as well as in exhaust gases of combustion processes and in atmospheric research. Optical measurement approaches may be based on light scattering [10], pyrometry (spectrally resolved [11, 12] or as a two-color method [11]), and light extinction [13]. While most of these techniques provide line-of-sight integrated data, other laser-based measurements, such as scattering, have been established for point-wise measurements and two-dimensional imaging. One of the most prominent of these approaches is laser-induced incandescence (LII) [14, 15], in which the aerosol particles are heated by a laser and information about the local volume fraction and particle sizes are determined from the intensity of the subsequent incandescence and its temporal variation.

While other laser-based measurements, including elastic (Rayleigh/Mie) scattering [16], inelastic (Raman) scattering [17], and photoluminescence (PL) [18, 19] (where the emitted signal results from a resonance between the materials and the excitation radiation) are linear processes with respect to laser fluence, the LII signal intensity has a strongly non-linear relationship with temperature and laser fluence [20], and only becomes significant above a material specific fluence threshold. At even higher fluences (generally above 100 mJ/cm^2^ for soot, with variations depending on the experimental setup [21]), particles can also partially or completely evaporate, which leads to a decline in LII intensity but opens additional pathways to characterize the particles from the gas phase or plasma, namely laser-induced breakdown spectroscopy (LIBS) [22] or resonant excitation of the generated gas-phase species [23] (as will be discussed in Sect. 7). At a given fluence, several of these effects can be active in parallel, depending on the material.

Soot is a particularly suitable material for LII, because most carbon structures do not sublime until above 4000 K, producing strong incandescence signals even in the presence of flame emissions. On the other hand, the models used to analyze LII measurements on soot rely on parameters, such as the absorption and scattering cross-section, density, specific heat, etc., which may not be known with a high degree of certainty. For example, the composition and structure depend on fuel composition and local stoichiometry, and evolve as the soot “ages” [15]. A further complicating factor concerns how desorbed species like polyaromatic hydrocarbons (PAH) affect the optical [24] and transport properties of soot [25].

To date, comparatively little effort has focused on deploying optical diagnostics, including LII, for characterizing inorganic “non-soot” nanoparticles in the gas phase, although this situation is rapidly changing, motivated by the unique properties of these materials and the economic importance of their large-scale production. From one viewpoint, applying LII to this class of materials presents several challenges beyond what are encountered when characterizing soot. The absorption cross-sections and maximum heat-up temperature (limited by sublimation, boiling, or decomposition) tend to be lower. The properties of some materials may be even less certain than those of soot (particularly materials having a heterogeneous structure or made of compounds), especially their radiative properties, densities, specific heats, evaporation enthalpies, vapor pressure functions over the temperature ranges pertinent to LII. On the other hand, non-soot nanoparticle targets are frequently elemental materials of known composition, and often have well-defined morphologies, e.g., isolated spherical nanoparticles having a narrow and tunable size distribution. In this respect, they represent ideal targets for LII. Measurements carried out on these materials can be used to validate and calibrate measurement models for soot and other materials having more ambiguous properties. In situations where the morphological and spectroscopic properties of the particles are well known, LII may also be used to interrogate fundamental thermodynamic and transport properties, such as those that define evaporation characteristics and thermal accommodation coefficients, which are otherwise difficult to measure under LII-relevant conditions. In other scenarios, LII measurements may be used to understand how pulsed lasers interact with complex materials, e.g., for plasmonic nanoparticles.

This paper has been invited in the context of our previous 2006 paper “Laser-induced incandescence: recent trends and current questions” [26] being identified as one of the top cited papers in the history of Applied Physics B. The recognition received by the 2006 paper, along with a 2007 model review paper by Michelsen et al. [27] and a 2015 review paper by Michelsen et al. [15], highlight the growing popularity of LII, particularly as a combustion diagnostic for characterizing soot nanoparticles. While these review papers almost exclusively focus on soot, the present review targets the rapidly growing field of non-soot LII, which, as already noted, involves aspects markedly different from LII on soot particles. Our review mainly concerns gas-borne engineered nanoparticles including non-carbonaceous particles and carbon allotropes with structures significantly different than soot (i.e., nanotubes, graphene, nanodiamonds). Because of its similarity to soot, (manufactured) carbon black is only briefly discussed, for comparative reasons. LII of particles that form in the atmosphere through condensation or through modification of emitted particles are not covered.

The current review focuses on LII using pulsed lasers. Continuous wave (cw) lasers have also been used for LII [28], but the measurement procedure is quite distinct from pulsed-laser LII. Continuous laser measurements are typically used in atmospheric science, while in situ measurements during and immediately after particle generation (including measurements at elevated ambient temperatures) are mostly based on pulsed laser excitation. As such, we do not consider applications of continuous lasers in this review.

The remainder of the paper begins with a brief overview of the spectroscopic (Sect. 2.1) and heat transfer (Sect. 2.2) models that underlie quantitative LII measurements, data analysis techniques (Sect. 2.3–2.4), and a brief discussion of some non-incandescent phenomena that may interfere with the LII signal. We next discuss LII measurements for various classes of nanomaterials, including elemental materials (metals and metalloids, Sect. 3), oxide and nitride materials (Sect. 4), carbonaceous non-soot materials (Sect. 5), and complex nanomaterials (Sect. 6). The paper concludes with a discussion about how LII can be complemented with other measurement modalities to reduce the uncertainty in inferred parameters (Sect. 7), as well as a general outlook into this emerging and exciting field.

## LII basics

**Fig. 1:**
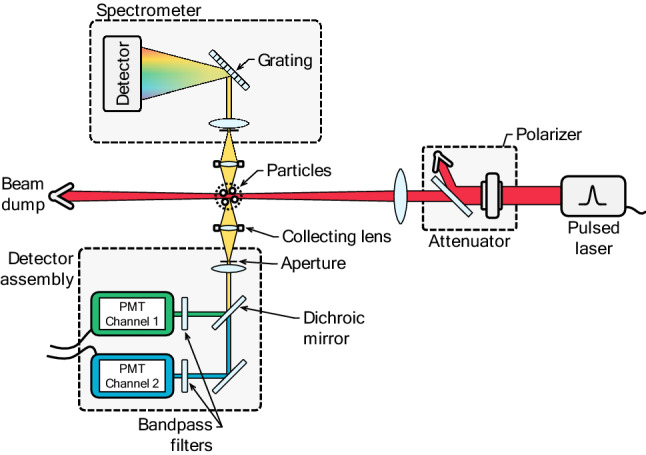
Schematic of a typical setup for time-resolved LII detection (TiRe-LII), including a two-color photomultiplier assembly, a spectrometer, and an attenuator to control the laser fluence, in this case via a polarizer and a half-wave plate. Setups for planar (e.g., [31–33]) and volumetric LII detection also exist in the literature but have been rarely used in non-soot LII scenarios to date

A typical LII setup is shown in Fig. 1 and was discussed in some detail by, e.g., Michelsen et al. [15]. The system consists of two subsystems: a pulsed laser and associated optics for exciting the nanoparticles in the aerosol probe volume; and a system for detecting the consequent laser-induced emission, usually at multiple wavelengths and often with high temporal resolution. The excitation system typically includes a Nd:YAG laser and optics needed to focus the beam. It is also commonplace to condition the beam to produce a time-averaged spatially uniform fluence profile, e.g., through relay-imaging [29, 30], and also to vary the laser fluence. While some studies have considered laser sheets for two-dimensional imaging (e.g., [31–33]) or even volumetric LII [34–36], these configurations have so far been used very rarely for LII measurements of non-soot nanoparticles, despite the potential utility of this sort of information, e.g., for understanding nanoparticle formation in synthesis reactors with turbulent flows [37].

The laser-induced emission from the probe volume is then imaged onto a detector system. Most often, the signal is measured at multiple wavelengths, either using photomultiplier tubes (PMTs) equipped with bandpass filters, or by dispersing the radiation and then imaging it onto a streak camera. Filtered PMTs provide high temporal resolution of the signal detection over a narrow spectral interval, and configurations using two or more (for enhanced dynamic range [38] or additional spectral information [39–41]) PMTs have been used to carry out LII on synthetic nanoparticles. On the other hand, gated spectrometers provide a spectrally resolved measurement at a particular instant, while spectrometer/streak camera configurations can provide both spectrally and temporally resolved signals, albeit with a lower time resolution compared to PMTs. Spectral resolution is particularly important in the case of LII measurements on synthetic nanoparticles for two reasons: the radiative properties of these nanoparticles often depend strongly on wavelength, in contrast to soot, and a spectrally resolved signal can reveal non-incandescence emission, such as excited-state emission from vaporized species, chemiluminescence, particle photoluminescence, particle plasmon resonances [42], or plasma that, if unnoticed, could interfere with bandpass-filtered detection. The details of the experimental configurations are both application and materials specific, for instance depending on whether measurements are made inside a nanoparticle reactor or on the exhaust flow of the reactor. As such, we often refer the reader to cited studies for experimental details.

The quantities to be inferred (i.e., the quantities-of-interest, QoI) are connected to the observed spectral incandescence signals through a measurement model composed of two coupled submodels: a spectroscopic submodel that relates the observed incandescence to the temperature of the nanoparticles within the probe volume at any given instant, and a heat transfer submodel that describes how the temperature of an individual nanoparticle changes during the measurement. The models used for non-soot particles share a common structure to those for soot, differing mostly in terms of the thermophysical and optical properties. However, these differences can yield very different peak temperatures, may be influenced by different phase transitions, and may determine whether the Rayleigh approximation can be used in the spectroscopic submodel.

### Spectroscopic submodel

In most time-resolved LII (TiRe-LII) experiments, the particles are modeled as spheres having diameters that obey a probability density function *p*(*d*_p_). The spectral intensity incident on the detector at any instant, *J*_λ_(*t*), is given by

1 $$J_{\lambda } \left( t \right) = \Lambda \int\limits_{0}^{\infty } {C_{{{\text{abs}},\lambda }} \left( {d_{{\text{p}}} } \right)I_{{\lambda ,{\text{b}}}} \left[ {T_{{\text{p}}} \left( {d_{{\text{p}}} ,t} \right)} \right]p\left( {d_{{\text{p}}} } \right){\text{d}}d_{p} } ,$$

where *C*_abs,λ_ is the spectral absorption cross-section of particles having a diameter *d*_p_, *I*_λ,b_ is the blackbody spectral intensity for particles at a temperature *T*_p_(*d*_p_, *t*), and Λ is a so-called intensity scaling factor, which accounts for the particle number density and detection solid angle and, in principle, is roughly proportional to the instantaneous volume fraction [43]. While some researchers have expressed the emitted incandescence in terms of emissivity [15], use of the bulk material quantity is conceptually incorrect for nanoparticles and should be avoided. This is principally because nanoparticles absorb and emit electromagnetic waves volumetrically, rather than as a surface. Thus, under some circumstances, the absorption efficiency of a nanoparticle can be greater than unity, leading in turn to an emissivity greater than unity. This observation is inconsistent with the macroscopic interpretation that the emissivity represents the ratio of emitted radiation relative to Planck’s distribution.

Spectral intensity at multiple wavelengths is usually merged into an instantaneous effective temperature, *T*_p,eff_(*t*), which provides some indication of the sensible energy of the nanoparticles within the probe volume at any instant and reduces the dimension of the input data. Most often, *T*_p,eff_ is calculated by modeling the particle sizes as having some representative uniform diameter *d*_p,eff_ [44] and by invoking Wien’s approximation, so that Eq. (1) may be rearranged into an explicit expression for temperature. Since Λ is, in principle, independent of the wavelength, it can be eliminated by combining spectral intensities measured at two detection wavelengths

2 $$T_{{\text{p,eff}}} \left( t \right) = \frac{{\frac{{hc_{0} }}{{k_{{\text{B}}} }}\left( {\frac{1}{{\lambda_{1} }} - \frac{1}{{\lambda_{2} }}} \right)}}{{\ln \left[ {\frac{{J_{\lambda 1} }}{{J_{\lambda 2} }}\frac{{C_{{{\text{abs}},\lambda 2}} \left( {d_{{{\text{p}},{\text{eff}}}} } \right)}}{{C_{{{\text{abs}},\lambda 1}} \left( {d_{{{\text{p}},{\text{eff}}}} } \right)}}} \right]}}.$$

Time-resolved experiments in which incandescence is measured at three or more wavelengths (e.g., using a streak camera [41, 45, 46]) provide more information and are less susceptible to emission artifacts. In this case, Λ and *T*_p,eff_ can be solved simultaneously through nonlinear regression. This calculation requires some prior knowledge of the particle diameter, which can be problematic in TiRe-LII experiments that have the objective of determining particle size, although it will be shown that this requirement can be relaxed under certain conditions.

Accurate spectroscopic modeling hinges on calculating the spectral absorption cross-section of the nanoparticles. In the case of nanoparticles having homogenous composition, this depends on the size of the nanoparticle relative to wavelength, expressed in terms of the size parameter,

3 $$x_{{\text{p}}} = {{\pi d_{{\text{p}}} } \mathord{\left/ {\vphantom {{\pi d_{{\text{p}}} } \lambda }} \right. \kern-\nulldelimiterspace} \lambda };$$

the bulk electromagnetic properties of the nanoparticle material, as defined by the refractive index *m* = *n* + i*k* or complex permittivity, *ε* = *ε*_I_ + *iε*_II_; and the nanoparticle morphology.

In the case of spherical particles, the spectral absorption cross-section can be calculated from the extinction and scattering efficiencies,

4 $$C_{{{\text{abs}},\lambda }}^{{}} = \frac{{\pi d_{{\text{p}}}^{2} }}{4}Q_{{{\text{abs}},\lambda }} = \frac{{\pi d_{{\text{p}}}^{2} }}{4}\left( {Q_{{{\text{ext}},\lambda }} - Q_{{{\text{scat}},\lambda }} } \right),$$

which, in turn, are obtained using Mie theory,

5 $$Q_{{{\text{ext}},\lambda }}^{{}} = \frac{2}{{x_{p}^{2} }}\sum\limits_{s = 1}^{\infty } {\left( {2s + 1} \right){\text{Re}} \left( {a_{s} + b_{s} } \right)}$$

6 $$Q_{{{\text{scat}},\lambda }} \frac{2}{{x_{{\text{p}}}^{2} }}\sum\limits_{s = 1}^{\infty } {\left( {2s + 1} \right)\left[ {\left| {a_{s} } \right|^{2} + \left| {b_{s} } \right|^{2} } \right]}$$

where Re(·) returns the real component of a complex number. In Eqs. (5) and (6), *a*_s_ and *b*_s_ are the scattering coefficients given by

7 $$a_{{\text{s}}} = \frac{{{\mathbf{m}}\psi_{s} \left( {{\mathbf{m}}\,x_{{\text{p}}} } \right)\psi^{\prime}_{s} \left( {x_{{\text{p}}} } \right) - \psi_{s} \left( {x_{{\text{p}}} } \right)\psi^{\prime}_{s} \left( {{\mathbf{m}}\,x_{{\text{p}}} } \right)}}{{{\mathbf{m}}\psi_{s} \left( {{\mathbf{m}}\,x_{{\text{p}}} } \right)\psi^{\prime}_{s} \left( {x_{{\text{p}}} } \right)\xi^{\prime}_{s} \left( {x_{{\text{p}}} } \right) - \psi_{s} \left( {x_{{\text{p}}} } \right)\xi^{\prime}_{s} \left( {{\mathbf{m}}\,x_{{\text{p}}} } \right)}}$$

and

8 $$b_{{\text{s}}} = \frac{{\psi_{s} \left( {{\mathbf{m}}\,x_{{\text{p}}} } \right)\psi^{\prime}_{s} \left( {x_{{\text{p}}} } \right) - {\mathbf{m}}\psi_{s} \left( {x_{{\text{p}}} } \right)\psi^{\prime}_{s} \left( {{\mathbf{m}}\,x_{{\text{p}}} } \right)}}{{\psi_{s} \left( {{\mathbf{m}}\,x_{{\text{p}}} } \right)\xi^{\prime}_{s} \left( {x_{{\text{p}}} } \right) - {\mathbf{m}}\psi_{s} \left( {x_{{\text{p}}} } \right)\xi^{\prime}_{s} \left( {{\mathbf{m}}\,x_{{\text{p}}} } \right)}},$$

where ψ_*s*_ and ξ_*s*_ are Ricatti–Bessel functions of order *s*. While Eqs. (5)–(8) are developed for a homogeneous sphere, Mie theory can also be used to model the cross-sections of spherically symmetric core–shell structures, such as those in Sect. 6.

Physically, each nanoparticle can be envisioned as an ensemble of dipoles that interact with an imposed oscillating electromagnetic (EM) field (e.g., the laser pulse) and with each other. A large number of terms are often required in the summation to capture these complicated interactions, making this calculation slow and cumbersome. A special case occurs when *x*_p_ <  < 1 and ||**m** · *x*_p_||< < 1. When both of these conditions are met, all of the dipoles within the nanoparticle “see” the same EM field at any instant and oscillate in phase. Then, the Mie equations collapse into

9 $$Q_{{{\text{abs,}}\lambda }} = 4x_{{\text{p}}} E\left( {\mathbf{m}} \right) = - 4x_{{\text{p}}} {\text{Im}} \left( {\frac{{{\mathbf{m}}^{2} - 1}}{{{\mathbf{m}}^{2} + 2}}} \right),$$

where *E*(**m**) is the refractive index-dependent absorption function, and Im(·) returns the imaginary component of a complex number. This is the Rayleigh limit, or the electrostatic approximation. In this limit, the absorption efficiency scales with 1/*λ*, and the absorption cross-section is proportional to the nanoparticle volume, *C*_abs,*λ*_ ∝ *d*_p_^2^*x*_p_ ∝ *d*_p_^3^. Physically, since the dipoles are oscillating in phase, there is no interaction between the dipoles so the absorption cross-section is proportional to the number of dipoles contained within the nanoparticle.

The electrostatic approximation of the absorption cross-section can be separated into terms containing the particle diameter and the bulk electromagnetic properties of the nanoparticle material. Substituting this expression for *C*_abs,λ_ into Eq. (2) results in an equation for *T*_p,eff_, independent of the particle diameter:

10 $$T_{{\text{p,eff}}} \left( t \right) = \frac{{\frac{{hc_{0} }}{{k_{{\text{B}}} }}\left( {\frac{1}{{\lambda_{1} }} - \frac{1}{{\lambda_{2} }}} \right)}}{{\ln \left[ {\frac{{J_{{\lambda_{1} }} }}{{J_{{\lambda_{1} }} }}\frac{{\lambda_{2} E\left( {m,\lambda_{1} } \right)}}{{\lambda_{1} E\left( {m,\lambda_{2} } \right)}}} \right]}}.$$

This greatly simplifies analysis of TiRe-LII data since the spectroscopic and heat transfer submodels can be applied as two sequential and independent steps. Otherwise, an iterative approach may need to be taken, or an estimate for *d*_p,eff_ would need to be assumed, which diminishes the physical relevance of *T*_p,eff_.

**Fig. 2:**
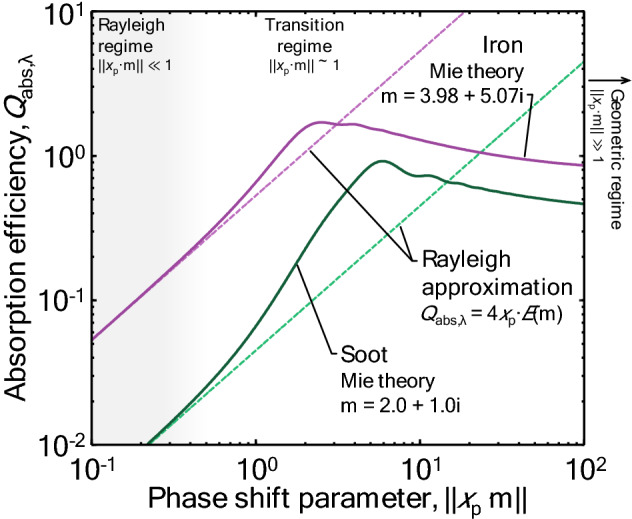
Absorption efficiency of an isolated sphere as a function of the size parameter, *x*_p_|**m**|, for iron and soot, showing the transition from the Rayleigh regime through the transition regime. Figure adopted from Ref. [47]. The grey shaded region to the left marks the Rayleigh regime

The conditions required for Eq. (9) to hold are usually satisfied for soot primary particles, so it is often the default spectroscopic model used when investigating the synthetic nanoparticles that are the focus of this paper. However, while most nanoparticles satisfy *x*_p_ << 1 for the wavelengths important to LII, the refractive index of metals is typically an order-of-magnitude larger than that of carbonaceous materials, like soot. As such, the requirement of ||**m** · *x*_p_|| << 1 is often not satisfied (Fig. 2). Not meeting this requirement does not significantly impact the accuracy of Eq. (2) [44], but it can affect some of the underlying assumptions about the distribution of nanoparticle temperatures in the probe volume as described later in this paper.

The increasing variety of synthetic nanoparticles analyzed using TiRe-LII is reflected both in nanoparticle materials as well as particle morphologies, which can include aggregates of spheres, prisms [48], core–shell structures (e.g., [49–51]) and more complex shapes, like few-layer graphene (FLG) flakes [41, 52]. As long as these particles absorb and emit in the Rayleigh regime, the absorption cross-section can usually be approximated as proportional to the volume/number of dipoles contained in the nanoparticle. For example, the absorption cross-section of soot aggregates is well-approximated by the sum of absorption cross-sections of individual primary particles through Rayleigh–Debye–Gans fractal aggregate theory [53], and preliminary research also suggests that the spectroscopic properties of FLG particles can be modeled using the electrostatic approximation [52].

**Fig. 3:**
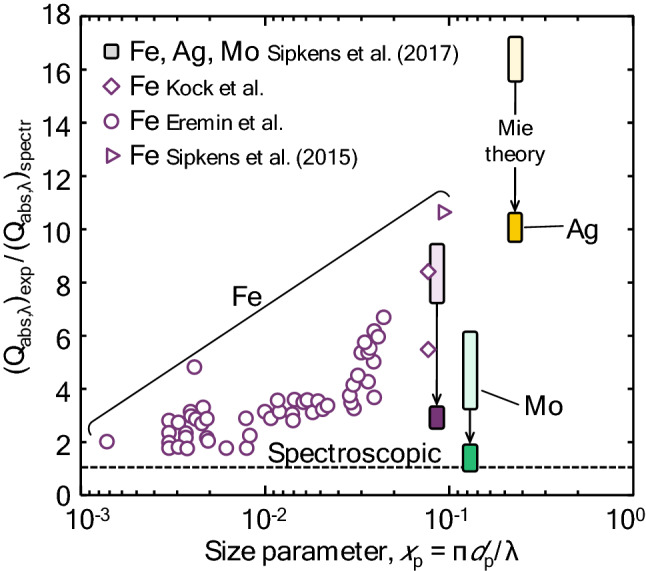
The ratio of the experimentally derived absorption efficiency (required to reach a pyrometrically inferred peak temperature) to the spectroscopic absorption efficiency (using Rayleigh theory and the complex index of refraction) from Kock et al. [58], Eremin et al. [59], and Sipkens et al. [60, 61] as a function of the size parameter. Results show excessive absorption, where the absorption efficiency required to reach the pyrometrically inferred peak temperature greatly exceeds that predicted using the Rayleigh approximation. Also shown are the values from Sipkens et al. [60] when predicting the absorption efficiency using Mie theory [44], which partially resolves the excessive absorption problem. For this latter case, the height of the shaded boxes corresponds to the range of ratios observed across the experimental conditions in that study

When the electrostatic approximation does not hold, phenomena such as excessive absorption (Fig. 3) may be observed. In this case, more sophisticated techniques must be adopted. As already noted, Mie theory can be applied to spherical core–shell structures, but for more complex shapes, numerical techniques like T-matrix [54, 55] and the discrete dipole approximation (DDA) [56] must be used to simulate the cross-section. While these techniques are highly accurate, they are also computationally intensive and require that the particle morphology be known a priori. Accordingly, if the particle size and morphology is to be inferred from the spectral incandescence measurements, it may be necessary to construct a “meta model” using principle component analysis [57] or a neural network [16] that can interpolate *C*_abs,*λ*_ from a dataset of values precomputed using a high fidelity model.

A further complication concerns the bulk refractive properties of the material. While these properties are well known for some pure materials (e.g., pure metals), in other materials they may not be as well-characterized. This is particularly the case for composite materials, where the absorption and extinction cross-sections will depend on the bulk properties of the component materials as well as their distribution within the nanoparticle. Also, the laser pulse may profoundly affect the absorption and extinction cross-sections of the nanoparticle, either by altering the structure of the nanoparticle (e.g., aggregate sintering [62]) or by changing the atomic and molecular bonding within the nanoparticle (e.g., melting [44, 63] or defect formation [64]). These changes are difficult to model or measure, and therefore, may significantly contribute to the overall uncertainty of the analysis.

### Heat transfer submodel

**Fig. 4:**
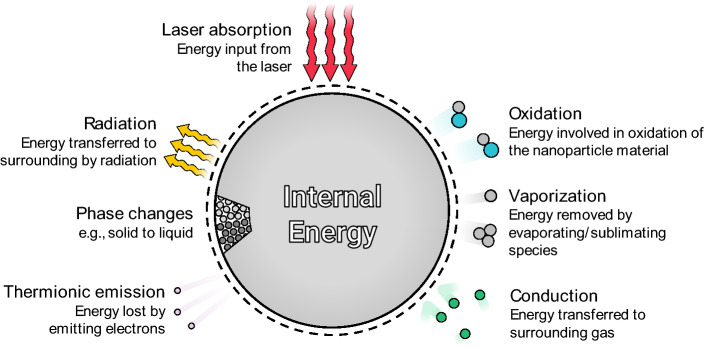
Schematic of the energy balance for an isolated primary particle, relevant to the TiRe-LII heat transfer model. In almost all scenarios, cooling is dominated by evaporation and conduction

Once the measured spectral incandescence is connected to the nanoparticle temperature through the spectroscopic submodel, the size-distribution parameters and other quantities-of-interest (QoI) may be inferred using a heat transfer submodel that predicts how the sensible energy of an individual nanoparticle, *U*_p_, evolves with laser heating and subsequent cooling according to the processes shown in Fig. 4. The temperature of a nanoparticle of diameter *d*_p_ is governed by

11 $$\frac{{{\text{d}}U_{{\text{p}}} }}{{{\text{d}}t}} = \frac{{{\text{d}}\left( {m_{{\text{p}}} c_{p} T_{{\text{p}}} } \right)}}{{{\text{d}}t}} \approx \rho c_{p} \frac{{\pi d_{{\text{p}}}^{3} }}{6}\frac{{{\text{d}}T_{{\text{p}}} }}{{{\text{d}}t}} = q_{{{\text{laser}}}} - q_{{{\text{evap}}}} - q_{{{\text{cond}}}} - q_{{{\text{other}}}} ,$$

where *ρ* and *c*_*p*_ are the density and specific heat of the nanoparticle temperature, *q*_laser_ is the energy added to the nanoparticle by the laser pulse, *q*_evap_ and *q*_cond_ are the rate of evaporative and conductive heat transfer from the nanoparticle, and *q*_other_ represents other cooling terms, such as thermal radiation and thermionic cooling. Under almost all TiRe-LII measurement conditions, these other cooling modes are at least an order-of-magnitude lower than conduction heat transfer, and are most often ignored. Equation (11) also neglects changes to the latent energy of the nanoparticle caused by rearrangement of the atoms and their bonds (e.g., due to annealing), although these are usually assumed to be small relative to the change in sensible energy.

The laser heating rate is given by

12 $$q_{{{\text{laser}}}} = F_{0} f\left( t \right)C_{{{\text{abs,}}\lambda {\text{laser}}}}$$

where *F*_0_ is the laser fluence (e.g., mJ/cm^2^), *f*(*t*) is a dimensionless temporal profile of the laser pulse, defined so that $\int_{0}^{\infty } {f\left( t \right){\text{d}}t} = 1$, and *C*_abs,λlaser,_ is the nanoparticle absorption cross-section at the laser wavelength. In most TiRe-LII experiments, *f*(*t*) has a temporal Gaussian profile with a full-width half-maximum (FWHM) on the order of ~ 10 ns, although picosecond pulse experiments have been carried out as well [65]. Equation (12) also assumes that the laser fluence is spatially uniform, i.e., a “top-hat” profile; otherwise it is necessary to account for spatial variation in the fluence. It is also important to note that spatially top-hat and temporally Gaussian profiles may only be achieved through the average of many laser pulses, and individual laser pulses have spatial and temporal profiles that deviate from these idealizations [66].

Evaporative and conductive heat transfer usually occur in the free molecular regime, in which molecules travel between the nanoparticle surface and the equilibrium gas without undergoing intermolecular collisions. This condition holds as long as the molecular mean free path in the gas is equal to or larger than the nanoparticle diameter [67]. In both cases, the heat transfer rate for spherical particles can be written as the product of the particle surface area, π*d*_p_^2^, a molecular number flux at the nanoparticle surface, and the average energy transfer per molecule.

Evaporation heat transfer is calculated by assuming that the condensed-phase and gas-phase material on either side of the nanoparticle interface is in equilibrium. Under these conditions, the evaporative heat transfer rate is

13 $$q_{{{\text{evap}}}} = \pi d_{{\text{p}}}^{2} N^{\prime\prime}_{{\text{v}}} \frac{{\Delta H_{{\text{v}}} }}{{N_{{\text{A}}} m_{{\text{v}}} }} = \pi d_{{\text{p}}}^{2} \frac{{n_{{\text{v}}} c_{{\text{v}}} }}{4}\frac{{\Delta H_{{\text{v}}} }}{{N_{{\text{A}}} m_{{\text{v}}} }} = \pi d_{{\text{p}}}^{2} \frac{{n_{{\text{v}}} c_{{\text{v}}} }}{4}\Delta h_{{\text{v}}} ,$$

where *N*_v_″ is the number flux of evaporated molecules, *n*_v_ and *c*_v_ are the number density and mean thermal speed of evaporated molecules, *m*_v_ is the molecular mass of the evaporated species, *N*_A_ = 6.02 × 10^26^ kg/kmol is the Avogadro number, and Δ*H*_v_ and Δ*h*_v_ are the molar and specific latent heats of vaporization, respectively. The mean thermal speed is given by *c*_v_ = [8*k*_B_*T*_p_/(π*m*_v_)]^1/2^ and the number density of evaporated species is

14 $$n_{{\text{v}}} = {{p_{{\text{v}}} } \mathord{\left/ {\vphantom {{p_{{\text{v}}} } {k_{{\text{B}}} T_{{\text{p}}} }}} \right. \kern-\nulldelimiterspace} {k_{{\text{B}}} T_{{\text{p}}} }},$$

where *k*_B_ is the Boltzmann constant and *p*_v_ is the vapor pressure.

From a thermodynamics perspective, TiRe-LII amounts to perturbing a system initially at thermal equilibrium with a laser pulse, and then measuring how quickly the system returns to equilibrium through heat transfer between the nanoparticles and the surrounding gas. While the microsecond time-scales typical of the nanoparticle cooling rate are much longer than the nanosecond-scale laser pulse, the sub-femtosecond timescales important to the phase equilibrium across the solid–liquid and vapor interface are much shorter [68], and therefore evaporative models like the Clausius–Clapeyron equation,

15 $$p_{{\text{v}}} = p_{{{\text{ref}}}} \exp \left[ { - \frac{{\Delta H_{{\text{v}}} }}{R}\left( {\frac{1}{{T_{{\text{p}}} }} - \frac{1}{{T_{{{\text{ref}}}} }}} \right)} \right],$$

which relies on the presumption that the Gibbs free energy is the same on either side of the phase interface, accurately predict the vapor number density at the particle surface for the purposes of modeling evaporative cooling.

For small nanoparticles (e.g., *d*_p_ < 10 nm), it may also be necessary to account for the increased energy of the curved interface via the Kelvin [69] and Tolman [70] equations, as in Refs. [46, 60, 61, 71–75] for metal and metalloid nanoparticles. A “sticking coefficient” is sometimes incorporated into Eq. (13) to account for evaporated molecules that may re-condense onto the nanoparticle; however, since the velocities of evaporating molecules follow a Maxwell–Boltzmann distribution at *T*_p_ away from the nanoparticle, only a very small fraction of evaporated molecules may be expected to return to the nanoparticle surface so this parameter is usually neglected. These effects remain uncertain [60, 74].

The conduction heat transfer rate is given by

16 $$q_{{{\text{cond}}}} = \pi d_{{\text{p}}}^{2} N^{\prime\prime}_{{\text{g}}} \left\langle {E_{{\text{g,o}}} - E_{{\text{g,i}}} } \right\rangle = \alpha \pi d_{{\text{p}}}^{2} \frac{{n_{{\text{g}}} c_{{\text{g}}} }}{4}\left\langle {E_{{\text{g,o}}} - E_{{\text{g,i}}} } \right\rangle_{\max } ,$$

where *N*_g_″ is the number flux of incident gas molecules, *n*_g_ = *p*_g_/(*k*_B_*T*_g_) and *c*_g_ = [8*k*_B_*T*_g_/(π*m*_g_)]^1/2^ from the equilibrium bath gas at *p*_g_ and *T*_g_, and a molecular mass *m*_g_, and <*E*_g,o_–*E*_g,i_> is the average energy transferred when a gas molecule scatters from the nanoparticle surface. This last term is written in terms of the thermal accommodation coefficient, *α*, which specifies the average surface energy transferred to a gas molecule relative to the maximum value allowed by the 2nd Law of Thermodynamics

17 $$\left\langle {E_{{\text{g,o}}} - E_{{\text{g,i}}} } \right\rangle_{\max } = \frac{1}{2}k_{{\text{B}}} \left( {4 + \zeta_{{\text{int}}} } \right)\left( {T_{{\text{p}}} - T_{{\text{g}}} } \right),$$

where ζ_int_ is the number of internal energy modes of the gas molecule. Given the brevity of the interaction between the gas molecules and the particle surface, the vibrational modes of the gas molecule can be usually be neglected, so ζ_int_ only accounts for the rotational degrees-of-freedom (zero for monatomic, one for diatomic and linear polyatomic, and two for nonlinear polyatomic). Some studies have instead phrased Eq. (17) in terms of the temperature-dependent specific heat ratio (e.g., from Filippov and Rosner [76]). This approach is not recommended as it may result in nonphysical trends in the data.

In contrast to refractory materials like soot, the comparatively low boiling point of other types of materials, such as metals (excluding molybdenum and tungsten) and semiconductors, leads to large evaporation rates. In these scenarios, it may be necessary to model the evolving mass of the nanoparticle according to

18 $$\frac{{{\text{d}}m_{{\text{p}}} }}{{{\text{d}}t}} = - \frac{{q_{{{\text{evap}}}} \left[ {T_{{\text{p}}} \left( t \right)} \right]}}{{m_{{\text{v}}} N_{{\text{A}}} \Delta H_{{\text{v}}} }}.$$

Equations (11) and (18) are coupled first-order ordinary differential equations that must be solved from initial conditions: typically the nanoparticles are initially at thermal equilibrium with the bath gas, *T*_p,i_ = *T*_g_, and *m*_p,i_ = *ρ*π*d*_p,i_^3^/6. These equations are coupled by the evaporation term in Eq. (18) and the instantaneous nanoparticle diameter, *d*_p_ = [6*m*_p_/(π*ρ*)]^1/3^, which affects the heat transfer terms in Eq. (11). The changing mass also produces an additional term on the left hand side of Eq. (11), but this is ignored since *c*_*p*_*T*_p_d*m*_p_/d*t* ≪ *m*_p_*c*_*p*_d*T*_p_/d*t*. Similar statements may hold for significant changes to the specific heat capacity. For multicomponent particles, the components may also have different boiling points and emitted species upon thermal decomposition.

While this basic model provides a general basis for analysis, it also presents several key limitations. For instance, Eq. (13) assumes free-molecular conduction. However, if the mean free molecular path is smaller than the nanoparticle diameter, the influence of collisions between molecules in the gas phase must be accounted for using a transition-regime model, e.g., Fuchs’ boundary-sphere method [67]; while these models are straightforward for spherical particles, in the case of non-spherical particles, it is necessary to define an “equivalent diameter” that may not be obvious from the particle geometry.

Evaporation heat transfer introduces further complexities into the model, particularly for low boiling point materials. In contrast to heat conduction, which can be reasonably modeled assuming a stationary gas, evaporated species leaving the nanoparticle are highly nonstationary, and can produce a “snowplow effect”, wherein the evaporated species push the bath gas molecules away from the particle [77]. To date, there is no satisfactory analytical model that captures this behavior.

The situation is even more complicated for non-elemental materials, such as mixtures or solid solutions and oxides/nitrides. In the former case, individual components may have different boiling points and thus evaporate at different rates depending on temperature, while in the latter case gaseous species (e.g., O_2_, N_2_) can be emitted upon thermal decomposition. In addition to its impact on evaporative heat transfer (due to the thermochemistry of the related reactions), this phenomenon could also fundamentally change the radiative properties of the nanoparticles through phase changes and defect formation, while the evaporating species may also contribute to non-incandescent laser-induced emission [42].

### Inferring quantities-of-interest from TiRe-LII data

Using the measurement model to infer quantities-of-interest (QoI) from TiRe-LII data can be challenging, particularly in view of the model uncertainties described in the previous section. Typically, inference is performed via least-squares fitting, where the residual between the data, **b**, and its modeled equivalent, **b**^mod^(**x**), is minimized. The vector **x** contains the QoI, which could include the particle size distribution parameters as well as other parameters like the thermal accommodation coefficient (TAC; *α*). We denote this mathematically as

19 $${\mathbf{x}}_{{{\text{MLE}}}} = \mathop {\arg \min }\limits_{{\mathbf{x}}} \left\{ {\left\| {{\mathbf{L}}^{{\text{b}}} \left( {{\mathbf{b}} - {\mathbf{b}}^{{{\text{mod}}}} \left( {\mathbf{x}} \right)} \right)} \right\|^{2} } \right\},$$

where ||·|| denotes the 2-norm, **L**^b^ is a weighting matrix, and **x**_MLE_ is the maximum likelihood estimate of the QoI. The quantity **L**^b^ represents the Cholesky factorization of the inverse covariance matrix, and weights the data so the noisiest data (typically at longer cooling times) has the least influence on the QoI estimates. Setting **L**^b^ equal to the identity matrix is equivalent to unweighted nonlinear least-squares regression, but the corresponding minimizer of Eq. (19) cannot be considered a maximum likelihood estimate. The minimization in Eq. (19) can be achieved using the least-squares solvers in any number of software packages.

The data, **b**, may be a set of incandescence signal traces or pyrometrically inferred temperatures, e.g., via Eq. (2). In the former case, **x** would include the QoI as well as the intensity scaling factor, Λ, from Eq. (1). In principle, this parameter should be time-independent provided that the particle volume fraction within the probe volume remains unchanged (i.e., the overall mass of evaporated nanoparticle material is small). Accordingly, any temporal variation in this parameter is an indication of a deficiency in the model [43] (see also discussion about apparent volume fraction and related anomalies in Refs. [30, 46, 78–82]). The main drawbacks of solving for Λ at every instant are the increase in computational effort and the increase in the statistical degrees-of-freedom, which increases the uncertainty of the recovered parameters.

On the other hand, working with pyrometric temperatures involves fewer data points, making the minimization problem computationally easier to solve. Moreover, the effective temperature may provide some physical insight into the average sensible energy within the probe volume at any instant. However, the effective temperature is also an implicit function of the particle size distribution, so modeling in this case requires a procedure of: (i) simulating the temperature at multiple sizes, (ii) computing the incandescence, (iii) integrating the incandescence over the size distribution, and, finally, (iv) calculating a modeled effective particle temperature from the integrated incandescence. We refer the reader to Ref. [44] for more information. Fitting to temperatures also requires some amount of data pre-processing, complicating uncertainty quantification, particularly when inferring information about the optical properties, which then get incorporated into computing the “data”.

Further insight into the vaporization properties, density, specific heat capacity, optical properties, and even the gas temperature, may be obtained by examining how the peak temperature or incandescence vary as a function of laser fluence [21]. While quantitative examples exist for soot (e.g., [83, 84]), applications to non-soot particles remain largely qualitative [41, 46, 60, 64, 75, 85]. This type of data may be combined with time-resolved data to improve the robustness of the inferred parameters via the Bayesian inference procedure described in the next section.

### Uncertainty quantification (UQ)

The QoI inferred from LII data must be interpreted in the context of their uncertainty, which arises from measurement noise, uncertainty of the model parameters, the simplifications employed when deriving the measurement models, and the uncertainty in the physical processes they are meant to capture. This is particularly the case because the inference process described in Sect. 2.3 can be mathematically ill-posed [86, 87], which amplifies these uncertainties into large errors in the recovered variables. Ill-posedness must be addressed by incorporating additional information into the inference process, e.g., an assumed shape of the particle size distribution or a thermal accommodation coefficient derived through molecular dynamics. Early non-soot studies typically only considered uncertainties stemming from signal noise (e.g., [88]), including inference of discretized particle size distributions [89, 90] and the influence of multiparameter analysis [58]. For example, in the case of low-fluence LII studies in which evaporation has a negligible influence on particle cooling, the primary particle diameter and thermal accommodation coefficient appear as a product in Eq. (12), such that they cannot generally be inferred simultaneously [58, 60, 81]. Another critical aspect of uncertainty quantification involves identifying nuisance parameters, model inputs that are not the focus of the inference procedure, but are imperfectly known (e.g., the vaporization properties when inferring the primary particle size and thermal accommodation coefficient [46, 73]).

**Fig. 5:**
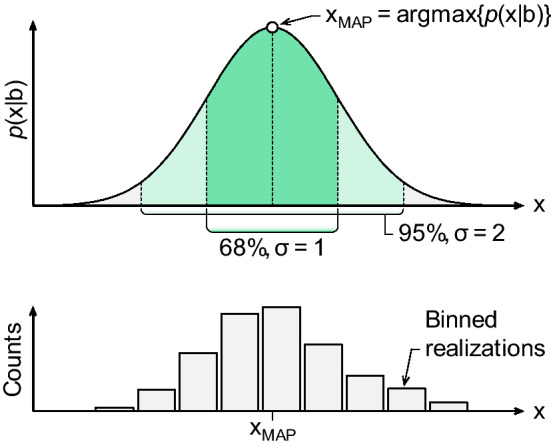
Schematics demonstrating the principle of random variables underlying the Bayesian framework, specifically for the posterior distribution. Quantities-of-interest, **x**, such as the primary particle diameter, are interpreted as random variables that follow some distribution (top). Repeated observations/experiments will result in different realizations that, when binned (bottom), will resemble these distributions. The distributions then contain information about estimates of the quantities, such as the maximum a posteriori (MAP) estimate, and corresponding uncertainties, shown here for a normal distribution

Traditionally, UQ in LII measurements has relied on perturbation analysis, in which one changes an input parameter by a pre-defined amount and computes the corresponding change in the inferred quantities [59, 81, 91]. However, a majority of UQ for non-soot TiRe-LII studies have employed the Bayesian framework, which instead treats all quantities (data, QoI, model parameters) as random variables defined by a corresponding probability density function (PDF), as shown schematically in Fig. 5. This treatment reflects the fact that none of these parameters are perfectly known, and the state-of-knowledge is best described by the width of a corresponding PDF. It is important to emphasize that the PDF does not necessarily represent the true distribution of the parameter but, rather, what is known about the parameter. If the parameter is well-known, the PDF may be nearly a delta-function, such that only one value is likely. By contrast, if little is known about the parameter, the PDF will be much flatter and wider, assigning non-zero probabilities to a wider range of parameter values. Treating quantities in this way allows for intuitive interpretation and treatment of uncertainties. Furthermore, one can formally introduce and weigh prior information via Bayes equation

20 $$p\left( {{\mathbf{x}}|{\mathbf{b}}} \right) \propto p\left( {{\mathbf{b}}|{\mathbf{x}}} \right)p\left( {\mathbf{x}} \right),$$

where *p*(**x**|**b**) is the posterior, the joint probability distribution for the inferred QoI; *p*(**b**|**x**) is the likelihood, derived from the data, measurement noise, and TiRe-LII model; and *p*(**x**) is the prior, containing any information known prior to the measurement, such as molecular dynamics (MD)-derived TACs along with their uncertainties. This approach was introduced by Sipkens et al. [72] for analyzing LII data from silicon nanoparticles and has subsequently been used for general UQ [61, 75], to incorporate nuisance parameters [46, 60], choose between thermophysical models [73], to combine data from multiple complementary diagnostics [60, 92], and to visualize limitations during inference [46, 60].

Often the likelihood and prior PDFs are modeled as Gaussian [72], having mean values that represent “best estimates” and covariances that reflect how well the values of these parameters are known. In this scenario, taking the logarithm of Eq. (20) amounts to an additional minimization term in Eq. (19) that promotes values for the QoI that conform with prior expectations. Uncertainties, meanwhile, can be calculated using

21 $${{\varvec{\Sigma}}}^{{{\text{po}}}} = \left[ {{\mathbf{J}}^{{\text{T}}} \left( {{{\varvec{\Sigma}}}^{{\text{b}}} } \right)^{ - 1} {\mathbf{J}} + \left( {{{\varvec{\Sigma}}}^{{{\text{pr}}}} } \right)^{ - 1} } \right]^{ - 1} ,$$

where **Σ**^b^ is the covariance of the data, often a diagonal matrix containing the square of the standard deviation in the measurement at each time in the signal; **Σ**^pr^ is the prior covariance, containing pre-determined uncertainties in the QoI or nuisance parameters, for instance, as summarized for the iron heat transfer model in Table 1 of [93]; **J** is the Jacobian, or sensitivity matrix, of the modeled data to the unknowns; and **Σ**^po^ is the covariance in the inferred quantities, which summarizes the uncertainty of the inferred quantities-of-interest (e.g., the diagonal contains uncertainties for each quantity). Equation (21) can be computed using matrix algebra in most programming languages (noting that the Jacobian is often an optional output from least-squares procedures). For more information, we refer the reader to the works cited at the end of the preceding paragraph, as well as dedicated works that describe the Bayesian framework [87, 94].

**Table 1:** Studies considering TiRe-LII from elemental particles, ordered by number of studies considering a given element

Element	Material class	LII studies	Boiling point [96]	Heat transfer model?
Iron (Fe)	Liquid phase metals	Vander Wal et al. [42], Starke et al. [91], Kock et al. [58], Eremin et al. [59, 71, 74, 97], Kiefer et al. [98], Gurentsov and Eremin [99], Sipkens et al. [60, 61, 73]	3134 K	Yes: see Table 2
Silicon (Si)	Liquid semiconductors (metalloids)	Eom et al. [100, 101], Sipkens et al. [72], Menser et al. [46, 92], Daun et al. [40]	3538 K	Yes: [46, 72, 92, 100, 101]
Molybdenum (Mo)	Refractory metals	Vander Wal et al. [42], Murakami et al. [88], Sipkens et al. [60, 81], Eremin and Gurentsov [85]	4912 K	Yes: [60, 81, 85, 88]
Silver (Ag)	Plasmonic nanoparticles	Filippov et al. [89], Sipkens et al. [60]	2435 K	No*
Nickel (Ni)	Liquid phase metals	Reimann et al. [102], Robinson-Enebeli et al. [75]	3186 K	Yes: [75]
Gold (Au)	Plasmonic nanoparticles	Talebi Moghaddam et al. [103]	3129 K	No
Tungsten (W)	Refractory metals	Vander Wal et al. [42]	5828 K	No
Titanium (Ti)	Refractory metals	Vander Wal et al. [42]	3560 K	No
Tin (Sn)	Liquid phase metals	Vander Wal et al. [42]	2875 K	No
Copper (Cu)	Liquid phase metals	Daun et al. [40]	2835 K	No
Germanium (Ge)	Liquid semiconductors (metalloids)	Menser et al. [92]	3106 K	Yes: [92]

*While Sipkens et al. [60] developed a heat transfer model for silver, recent work [44] has cast doubt on its utility, as the pyrometrically inferred temperatures in this study are unlikely to correspond to the true temperature because of potential interference from emission other than LII (see Sect. 3.2).

**Table 2:** Comparison of the heat transfer models for iron, the most studied of the elemental particles

Property	Starke [91]^‡^	Kock [58]	Eremin–Gurentsov [59, 71, 74, 97, 99]	Sipkens–Singh [60, 61]	Sipkens–Hadwin [73]*
Density, *ρ* (kg/m^3^)	7700	7700	8200–0.6 T/K Ref. [97] uses 7700	8171–0.650 T/K [124]	8171–0.650 T/K [124]
Specific heat capacity, *c*_p_ (J/kg·K)	650 [125]	824 [125]	*f*(*T*), piecewise smooth [125]	835 [123]	*f*(*T*), piecewise linear [123]
Thermal accommodation coefficient, *α*	0.33 (Ar)	0.13 (Ar) 0.13 (N_2_)^†^	0.01 (He) 0.1 (Ar) 0.13 (CO)	Typically inferred in a specific study	0.236 (Ar)
Eq. degrees of freedom, (4 + ζ_int_)	4 (only Ar)	4^†^	(1 – γ) / (1 + γ)	4 + ζ_int_	4 (only Ar)
Heat of vaporization, Δ*H*_v_ (kJ/mol)	–	375.8	375.8	Watson eq	Román eq
Vapor pressure, *p*_v_ (kPa)	–	Clausius–Clapeyron eq *p*_ref_ = 3.337 kPa *T*_ref_ = 2500 K [96]	Clausius–Clapeyron eq *p*_ref_ = 3.337 kPa *T*_ref_ = 2500 K [96] Ref. [71] includes Kelvin eq	Clausius–Clapeyron eq *p*_ref_ = 101.3 kPa *T*_ref_ = 3134 K Δ*H*_v,ref_ = 6090 kJ/mol [126], includes Kelvin eq	Clausius–Clapeyron eq *p*_ref_ = 101.3 kPa *T*_ref_ = 3073 K Δ*H*_v,ref_ = 6571 kJ/mol [126], includes Kelvin eq
Surface tension, [N/m]	–	–	2.40–2.85 · 10^–4^ T/K [127]	*f*(*T*) [128]^*i*^	1.865 [128]

Temperatures are in Kelvin. The surface tension contributes to the evaporation submodel whenever the Kelvin equation is implemented for the vapor pressure. Equivalent (eq.) degrees-of-freedom refers to the quantity (4 + ζ_int_) in Eq. (17). The quantity *γ*, relevant to the Eremin–Gurentsov model, refers to the ratio of the specific heat capacities of the gas, whose value was not explicitly stated in those works. Thermal accommodation coefficients were typically inferred and then used in subsequent studies

^†^Note that in the absence of accounting for the internal degree of freedom for *N*_2_, the Kock model thermal accommodation coefficient will not match the corresponding physical quantity (correction results in α = 0.09)

^‡^Starke model was developed only for low fluences

^*^Sipkens–Hadwin model is the optimal model of those presented in that work, as chosen via Bayesian model selection

^*i*^*f*(*T*) = 1.865 – (*T*/K – 1823) · 0.35 · 10^−3^

Overall, uncertain model parameters have repeatedly been shown to be the main drivers of uncertainties in the QoI, whether this be by more traditional techniques [59, 61, 81, 91] or using Bayesian approaches [46, 60]. In fact, the uncertainties from an ensemble of nuisance parameters are often an order-of-magnitude larger than contributions from measurement noise, particularly when the parameters are correlated (e.g., uncertainties in inferred particle sizes increase from around 1.5% to more than 30% [60], where the latter better reflects the real uncertainties in repeated measurements).

## Elemental materials and alloys

LII studies on elemental nanoparticles have primarily focused on metals and metalloids, with the full list of materials shown in Table 1. For analyzing the LII signal, these materials present an advantage over other systems (e.g., soot) in that their composition, structure, and morphology are often well known, and may even be modified by adjusting their synthesis conditions. This enables a systematic analysis of all relevant particle properties, especially the mean particle diameter [95] and gas atmosphere [61], within which the particles are characterized, in sharp contrast to soot particles formed through combustion. Lessons learned from measurements on well-characterized elemental nanoparticles with carefully controlled boundary conditions can potentially be adopted to more complex scenarios, including LII measurements on soot. On the other hand, lower peak temperatures restricted by lower boiling points lead to less intense signals, and the often-unknown optical properties must be determined for each material to enable quantitative measurements (cf. Sect. 3.2). LII measurements on metal nanoparticles also have some unexpected spectroscopic phenomena that, in some cases, may confound interpretation of these results.

**Fig. 6:**
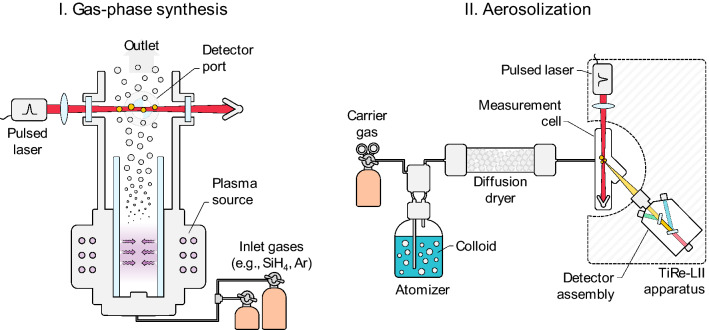
Examples of the two types of experiments: (I) characterization directly following gas-phase synthesis (in this case, in a plasma reactor), where the motive gas is constrained, or (II) characterization following aerosolization of a colloid (or powder, not shown) dispersed in a motive gas

Experiments broadly fall into two categories depending on how the test aerosol is produced: directly in the aerosol after particle formation, or via aerosolization of nanoparticles from a colloid or a powder. Examples of these scenarios are shown in Fig. 6. Aerosols of elemental nanoparticles may be generated directly through gas-phase reactions [104], including via plasma-assisted synthesis [105–107], thermal initiation in hot wall reactors [108] or shock tubes [109], flame synthesis, photolysis via UV lasers [88, 97], laser ablation [110], and arc discharge [40]. Plasma-assisted synthesis routes are particularly attractive, since they involve a well-defined reacting gas atmosphere and produce particles free of contaminants. Moreover, the particle size may be controlled by changing operating parameters like pressure, power, and flow rate [105]. Many scenarios also produce isolated spherical nanoparticles that obey a narrow size distribution, which is ideal for TiRe-LII. A drawback of gas-synthesis routes is that the gas composition influences both the particle size as well as the LII signal decay, which complicates comparison of LII measurements made in different atmospheres, and many synthesis processes work with only a limited range of gases (e.g., silicon experiments typically have some amount of hydrogen).

Alternatively, the aerosol may be generated indirectly from commercially available powders, which are dispersed using a drift tube [111] or a shock wave [89], or nanoparticle colloids using a pneumatic atomizer [60, 61]. These approaches can produce virtually any combination of nanoparticle and bath gas and, since the particle size does not depend on the bath gas, simplifies comparative analysis between gases. Such a setup is often less expensive, requiring less equipment. However, colloids must be sufficiently diluted to ensure that the majority of nanoparticles do not agglomerate, which can result in a low aerosol volume fraction and a weak LII signal, and, in most cases, a surfactant or polymer capping agent, which is needed to prevent aggregation in the colloid. While the capping agent is presumably ablated by the laser pulse, it nevertheless may influence the LII measurement.

**Fig. 7:**
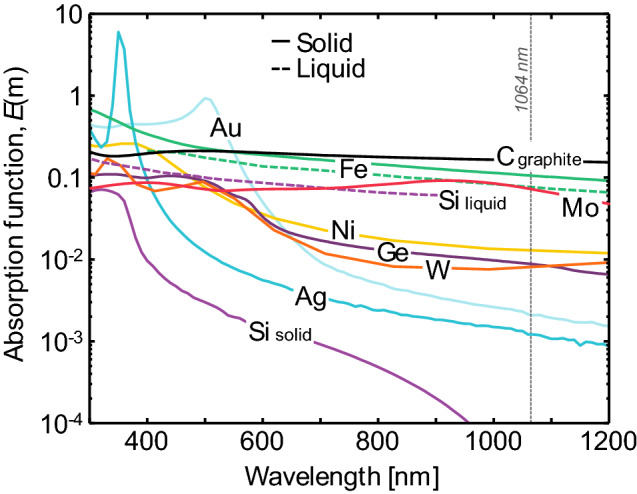
Measured (not from LII) absorption functions for a range of metals and metalloids as a function of wavelength. Solid lines correspond to properties for solids, while dashed lines correspond to liquids. The vertical scale is logarithmic, to emphasize differences in the absorption function in the near infrared. Note the difference between the absorption function of solid (semiconductor) and liquid silicon (metal). Underlying optical properties are taken from Schinke et al. [112] for solid silicon, Fuchs [113] for liquid silicon, Nunley et al. [114] for germanium, Querry [115] for molybdenum and solid iron, Shvarev et al. [116] for liquid iron, Werner et al. [117] for nickel and tungsten, McPeak et al. [118], for gold and silver, and Djurišić and Li [119] for graphite

**Fig. 8:**
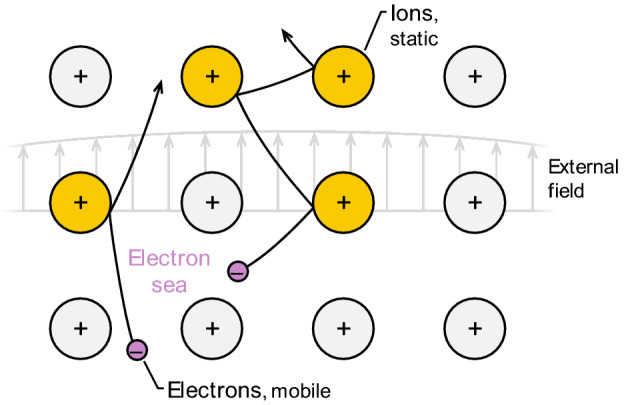
Schematic of a Drude metal. Free electrons accelerate and scatter off nearly static ions in the material when subjected to an external electromagnetic field. This is mechanism by which EM wave energy of the laser pulse is transferred to the internal energy of the nanoparticle. Figure adapted from Ref. [47]

As outlined in Sect. 2.1, the complex permittivity is a crucial component of the spectroscopic submodel. The radiative properties of some metals (e.g., silver, liquid silicon, liquid germanium) (Fig. 7) can be interpreted via Drude theory (Fig. 8), where the complex dielectric function is defined through the plasma frequency, *ω*_p_, and the mean collision time of conduction-band electrons, *τ*:

22 $$\varepsilon_{{\text{I}}} = 1 - \frac{{\omega_{{\text{p}}}^{2} \tau^{2} }}{{\omega^{2} \tau^{2} + 1}}$$

and

23 $$\varepsilon_{{{\text{II}}}} = \frac{{\omega_{{\text{p}}}^{2} \tau^{2} }}{{\omega \left( {\omega^{2} \tau^{2} + 1} \right)}}.$$

The complex dielectric functions of metals with interband transitions, as well as semiconductors, can be modeled using Drude–Lorentz theory

24 $$\varepsilon \left( \omega \right) = \varepsilon_{\infty } + \sum\limits_{i} {\frac{{f_{i} }}{{\omega_{i}^{2} - \omega^{2} - {\text{i}}\omega \Gamma_{i} }}} ,$$

where *f*_*i*_ is the oscillator strength, ω_*i*_ is the transition frequency, Γ_*i*_ is the linewidth for each transition within the spectral detection range, and *ε*_*∞*_ is the high frequency permittivity limit (accounting for core electrons). For semiconductors (e.g., silicon and germanium), these transitions are tabulated [120, 121].

**Fig. 9:**
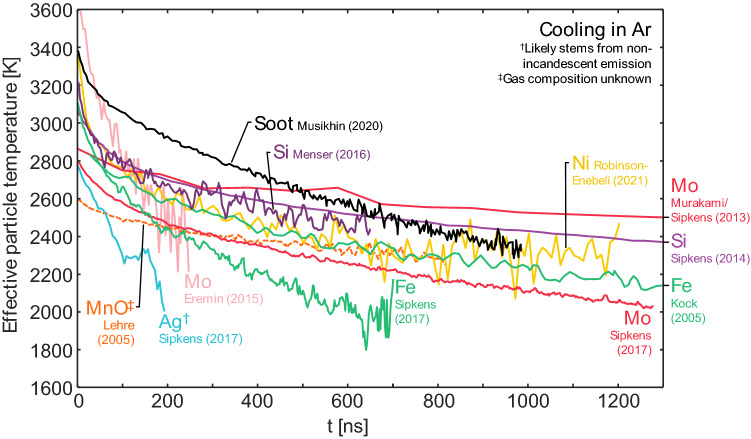
Measured effective temperature decay for a range of elemental nanoparticles in mostly argon environments (silicon and soot also had small amounts of hydrogen; also, see note on MnO). Data is from Kock et al. [58]; Murakami et al. [88] following reanalysis in Ref. [81]; Sipkens et al. [72]; Eremin and Gurentsov [85]; Menser et al. [46]; Sipkens et al. [60]; Musikhin et al. [41]; and Robinson-Enebeli et al. [75]. The Sipkens et al. (2017) and Robinson-Enebeli et al. studies had lower gas temperatures (298 K) than the other cases. The Eremin et al. and Robinson-Enebeli et al. studies have subsampled data—where every 4th or 25th data points were plotted, respectively—to make curves more distinguishable from one another while maintaining the magnitude of the measurement noise. ^†^The signals from silver particles are likely a different form of laser-induced emission, such that effective temperatures are likely non-physical [44]. ^‡^The gas composition for the single oxide (MnO) case shown (Lehre et al. [90]) was not stated

The unambiguous thermophysical properties of elemental nanoparticles (especially density and specific heat) make for a reliable heat transfer submodel, and these properties are available from various thermodynamic databases. Equilibrium thermodynamics also strongly favors the evaporation of single atoms from metals (e.g., [122, 123]) which is considerably simpler than for carbon allotropes, where a range of molecules may be expected depending on the nanoparticle temperature. Differences in material properties cause a larger range of potential temperature decays unique to a given material (Fig. 9). Heat transfer models have been developed for iron, silicon, molybdenum, nickel, and germanium (Table 1) (while a model was presented for silver [60], recent work has cast doubt on its applicability [44]). Models typically share a similar form, often differing only in terms of the material properties (cf., the differences between the four available models for iron in Table 2).

### Inferred quantities

Most TiRe-LII studies target aerosol attributes such as particle size and volume fraction, including some very early studies [89, 91, 100, 101]. For example, Filippov et al. [89] first attempted to apply the technique to infer particles sizes for silver nanoparticles, while Eom et al. [100] later mapped out the temporal growth of silicon nanoparticles in a plasma reactor.

**Fig. 10:**
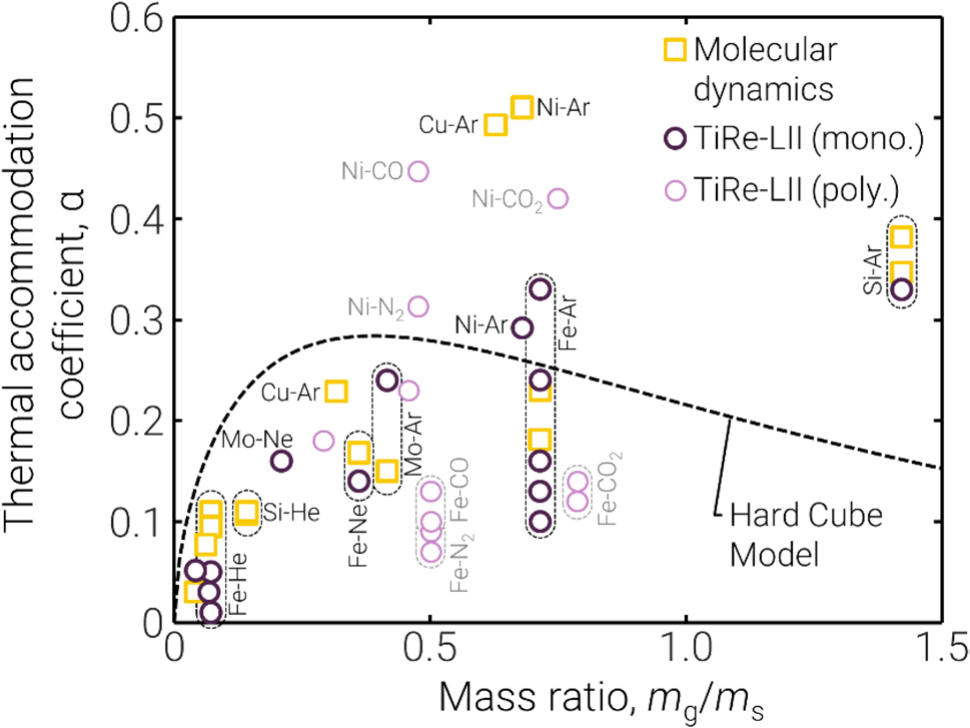
Trends in the thermal accommodation coefficient for a range of element-gas pairs with the ratio of the mass of the gas molecule, *m*_g_, to that of the nanoparticle material atomic mass, *m*_s_. Hard cube model prediction is described by Sipkens and Daun [132] for gas and surface temperatures of 300 K and 2500 K, respectively. Values are taken from Refs. [46, 58, 60, 61, 75, 91, 97] for the experimental TiRe-LII data and Refs. [72, 133–135] for the molecular dynamics data

Experimental conditions are sometimes sufficiently well-controlled to permit inference of more fundamental parameters that are otherwise difficult to measure through alternate means, particularly at the high temperatures typical of LII measurements. For example, during the development of the TiRe-LII technique, the thermal accommodation coefficient was usually treated as a “tuning parameter” that could be adjusted to “force” modeled cooling curves or TEM-derived particle sizes to match LII signal traces. TACs inferred in this way were confounded by model errors, and could not be interpreted in a strict physical sense. This situation has changed as the LII community has developed an increasingly detailed knowledge of the nanoparticle cooling mechanisms. In particular, a comparative analysis of TACs obtained from various elemental nanoparticle-gas combinations has provided key insights into the gas–surface scattering process underlying this parameter, including the influence of the mass ratio of the involved gas-phase molecules and surface atoms (Fig. 10), the gas–surface potential well, and the structure of the gas-phase molecule. Elemental particles simplify the assessment, as surface potentials are often better characterized for the elements than for multi-element compounds. In general, the TAC increases with molecular mass of the colliding gas-phase species according to a “square root” law, and the TAC for more structurally complex gas molecules is lower, since the surface energy of the particle is accommodated into the internal (rotational, vibrational) modes of the gas-phase molecule less efficiently [129–131]. Anomalies to this pattern may provide insights into unusual gas–surface interactions, e.g., for nickel [75].

**Fig. 11:**
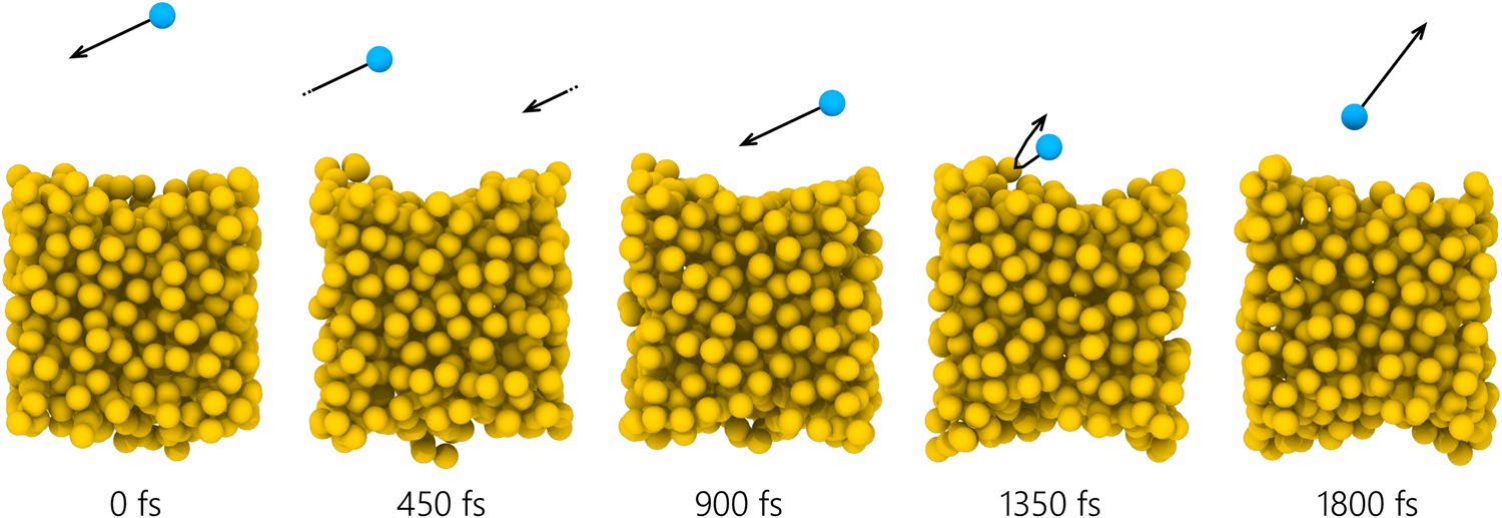
Scattering of a helium atom from a liquid silicon surface based on molecular dynamics simulations. Simulations such as this indicate that the TAC depends strongly on surface state. Adapted from Sipkens and Daun [135]

Kock et al. [58] and Eremin et al. [97] were the first researchers to examine how the TAC varies with bath gas for iron nanoparticles; subsequent studies consider iron [60, 61, 71], silicon [46, 72, 92], molybdenum [60], and nickel [75] nanoparticles in a range of monatomic and polyatomic gas-phase species. TACs derived from classical MD simulations [72, 133–135], Fig. 11, have often been compared with estimates from LII studies [60, 61, 72, 75], with consistencies observed across a wide range of particle-gas pairs. In the case of molybdenum nanoparticles, it is impossible to obtain robust estimates of particle size and thermal accommodation coefficient simultaneously [81], since evaporative cooling is negligible and the TAC and particle diameter terms appear as a product in the heat conduction term, cf. Eq. (16). Accordingly, Sipkens et al. [60] used a MD-derived TAC to infer particle sizes from LII measurements carried out on molybdenum nanoparticles aerosolized in argon.

Likewise, both the latent heat of vaporization and vapor pressure have been inferred for metal nanoparticles [71, 73, 74, 92]. Most often this is done in terms of the Clausius-Clapeyron or Antoine equation parameters. Eremin et al. [71, 74] attempted to infer size-dependent vaporization properties. Sipkens et al. [73] applied Bayesian model selection on TiRe-LII data collected on iron nanoparticles to identify the most probable model to account for the temperature-dependence of the latent heat of vaporization of iron. Menser et al. [92] carried out a combined TiRe-LII/LIBS measurement on liquid silicon nanoparticles, in which the evaporation rate was obtained directly from the intensity of an atomic emission line corresponding to evaporated silicon atoms. This measurement (discussed in greater detail in Sect. 7) represents the first independent validation of the standard LII evaporation model defined in Sect. 2.2.

### Fundamental challenges of measuring elemental nanoparticles

While LII studies on metal nanoparticles offer advantages over experiments on carbonaceous nanoparticles, there are also a number of key drawbacks. The signal-to-noise ratio of LII measurements largely depends on the maximum temperature to which the nanoparticles can be heated, which is governed by a balance between laser heating and cooling by evaporation or sublimation during the pulse [21]. In the case of soot, peak temperatures become limited by sublimation at 4200–4300 K, while peak temperatures of laser-heated metal nanoparticles are typically below 3000 K. According to Wien’s approximation, the spectral intensity at a given wavelength is proportional to exp(*−C*_2_/λ*T*) with *C*_2_ = *hc*/*k*_B_, so this difference in temperatures translates into a significantly lower intensity, and consequently a lower signal-to-noise ratio.

The situation is further confounded by the fact that the absorption cross-sections of metal nanoparticles, particularly those of high electrical conductivity, are much lower than carbonaceous nanoparticles. Combined, the lower evaporation temperatures and low absorption cross-sections of metal and solid metalloid nanoparticles result in a much weaker spectral intensity compared to what is typical for LII measurements on carbonaceous materials. Accordingly, significant experimental effort must be invested to provide highly efficient signal detection in order to achieve an acceptable signal-to-noise ratio. Of particular note, the weak absorption of metalloids at room temperature may limit studies at room temperature, with measurements easier when the particles start at an elevated temperature, e.g., in gas-phase synthesis reactors [46, 72]. Other means of increasing the signal are typical of LII experiments more broadly: more efficient optics; wider collection angles; multiple, gated PMTs to improve the dynamic range (see Ref. [38]); and wider bandpass filters. Q-switch noise from the pulsed laser may also corrupt fast-decaying signals, requiring special treatments to remove [75].

As noted in Sec. 2.1, a further complication is that the Rayleigh approximation of Mie theory cannot be applied to metal nanoparticles due to the fact that the phase shift criterion, ||**m**·* x*_p_||< 1, is not generally satisfied for metals. Consequently, the spectral absorption cross-section must be calculated using Mie theory [44, 136], or some higher order approximation for spheres [137]. Unfortunately, this calculation requires knowledge of the nanoparticle diameter, which is frequently unknown. In these scenarios, the Mie theory calculation must be repeated during each step of the optimization routine, which may require a large computational effort. Some discussion of this procedure can be found in [44] (A similar procedure must be followed if the Rayleigh approximation is invoked but the optical properties are either inferred or considered nuisance parameters, reevaluating the spectroscopic model during optimization, as per [60, 61]). In the case of LII experiments on soot, it is usually assumed that all the particles within the probe volume reach the same peak temperature, even for non-uniformly sized primary particles. This is because, in the case of the Rayleigh approximation, the absorption cross-section and the sensible energy are both proportional to volume. When the Rayleigh approximation does not apply, the particles do not all reach the same peak temperature, further complicating analysis [44]. For non-spherical nanoparticles, one would need to use advanced modeling techniques, e.g., T-matrix [54, 55] or discrete-dipole approximation (DDA) [56]. (The well-known Rayleigh–Debye–Gans fractal-aggregate model is based on the Rayleigh approximation for primary particles, and thus cannot be used for metal nanoaggregates). Unfortunately, many LII studies of metal nanoparticles have been carried out based on the faulty assumption that the Rayleigh approximation is valid, leading to erroneous results and conclusions.

Finally, there remain significant open questions about the spectroscopic submodel for certain metal nanoparticles. For example, studies have reported that the peak pyrometric temperatures of iron [59, 60] and silver nanoparticles [60] far exceeded that which should be possible based on the laser fluence and spectral absorption cross-section of the nanoparticles, although these analyses are flawed by the improper application of Rayleigh theory. Talebi Moghaddam et al. [44] reassessed these results using Mie theory and accounting for polydisperse particle sizes; while these modifications reduced the discrepancy for both materials, the effective absorption cross-sections for iron and silver nanoparticles remained two and ten times larger, respectively, than the values predicted using the dielectric functions of these materials. In principle, silver nanoparticles heated by a Nd:YAG laser pulse at 1064 nm, a duration of ~ 10 ns, and a fluence of ~ 100–200 mJ/cm^2^ should not exceed ~ 100 K above their initial temperature. This conflicts with the spectroscopically inferred temperatures around 1500 K reported in [44]. These observations strongly suggest that the laser absorption and emission models for these systems, as defined in Sect. 2.1, may be incomplete.

Talebi Moghaddam and Daun [138] evaluated the possibility that the observed LII signal from metal nanoparticles may, at least in part, consist of bremsstrahlung radiation from a laser-induced plasma enveloping the nanoparticle. Bremsstrahlung radiation is emitted as electrons decelerate around atoms and ions in the gas phase. These electrons may originate from thermionic emission by hot metal nanoparticles, which is plausible for iron nanoparticles [138]. The low absorption cross-section of silver and gold nanoparticles preclude any heating by the laser pulse; in this scenario the observed LII signal likely consists entirely of bremsstrahlung, and the electrons may originate from multiphoton-induced photoemission [103]. This hypothesis is supported by the variation of spectral intensity with fluence for aerosols of gold and silver nanoparticles within helium, neon, and argon irradiated by a 1064 nm laser pulse. More recently, Samuelsson et al. [139] described broadband photoluminescence from gold, silver, and copper nanoparticles irradiated by a continuous wave laser at 532 nm.

According to the Drude model, the EM wave of the laser interacts directly with conduction band electrons, which scatter from “background” ions in the metal. Consequently, in experiments involving short laser pulses, it is necessary to distinguish between the electron temperature and that of the ions, which lags according to the relaxation time, τ, in Eqs. (22) and (23) [140]. Since this timescale is on the order of 1 ps, it is generally assumed that the nanosecond timescales relevant to LII provide ample opportunity for the electrons and ions to equilibrate. Altman [141, 142] proposed an alternative hypothesis in which the electrons remain thermally isolated from the ions, and therefore the LII signal corresponds only to the electron temperature as opposed to that of the ensemble nanoparticle. This hypothesis relies on a theoretical study by Belotskii et al. [143] and appears to conflict with Drude theory. Further experimental work is needed to clarify the validity of this viewpoint.

## Oxide and nitride materials

According to Drude–Lorentz theory, oxides of light elements such as silicon, titanium, or aluminum couple with EM fields via bound charges that oscillate within the lattice. Therefore, in contrast to metals, oxide materials, such as SiO_2_, TiO_2_, and Al_2_O_3_, only weakly absorb in the visible and near infrared, making them inherently more challenging as LII targets. Their small absorption cross-sections make them difficult to heat, and also contribute to a weak incandescence signal that is susceptible to interference from other laser-induced emission sources. Due to these challenges, LII is used less frequently to characterize oxide (and nitride) nanoparticles compared to metal and carbonaceous nanoparticles, with some notable exceptions: in what is widely considered the first deployment of TiRe-LII, Weeks and Duley [111] targeted alumina particles irradiated with a pulsed CO_2_ laser.

In the case of transparent oxides like titania and silica (that appear white due to light scattering), laser heating and emission is expected to arise mainly through defects [64], which constitute localized charges in the lattice, and form in a way that depends on temperature, laser radiation, or as a consequence of kinetic effects during particle synthesis [144]. Alternatively, multiphoton interactions have been discussed as a way to overcome the wide band-gap [145], particularly for excitation with pulsed lasers and short excitation wavelengths. To improve heat-up efficiency during excitation, LII experiments on oxide nanoparticles are usually performed with visible or UV laser pulses.

**Fig. 12:**
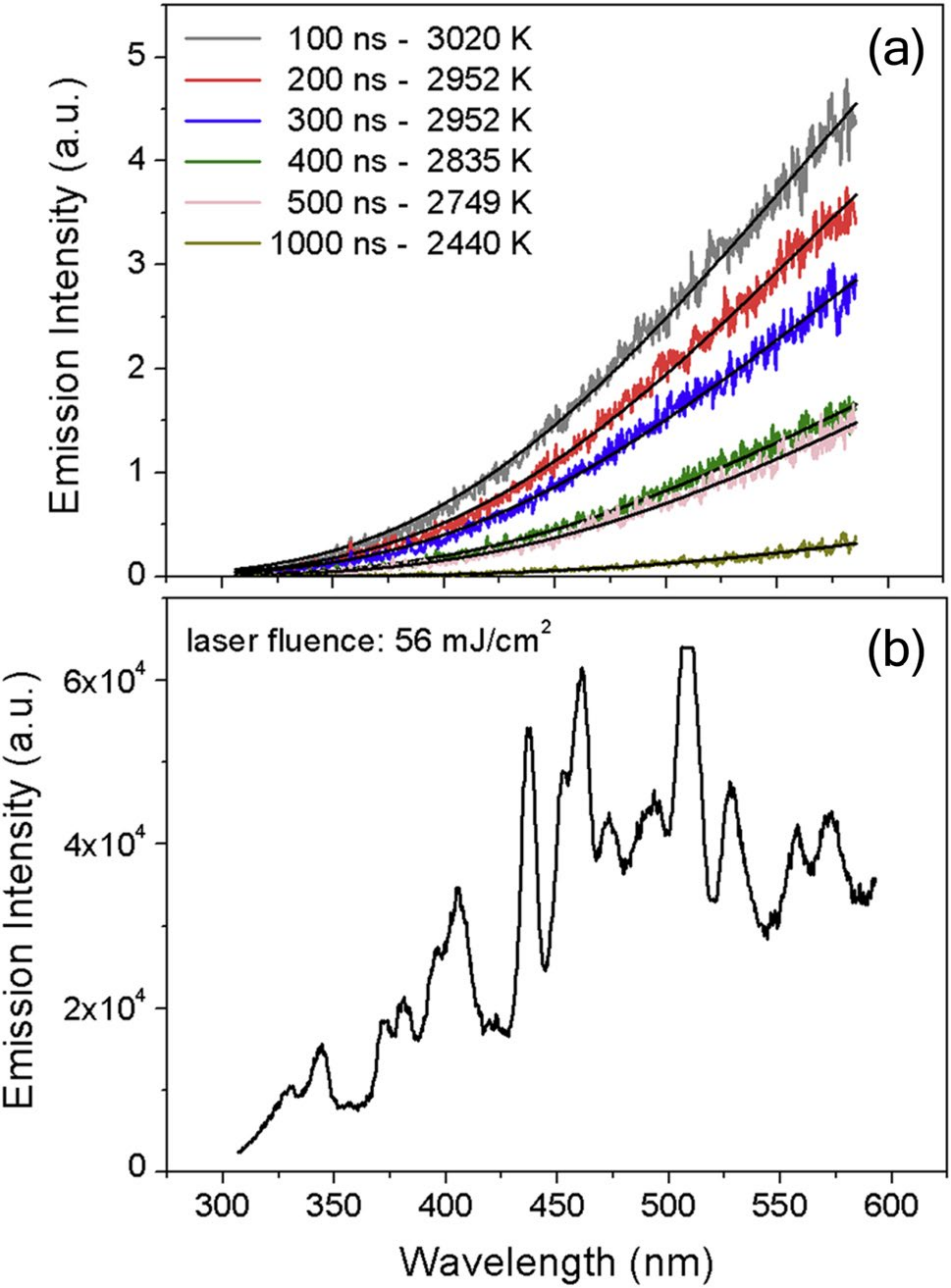
**a** Broadband laser-induced incandescence of TiO_2_ nanoparticles deposited on glass fiber filter with excitation at 266 nm at 17 mJ/cm^2^ laser fluence at various delay times with associated temperatures evaluated from fitting Planck functions. **b** The same sample with higher fluence (56 mJ/cm^2^) showing strong overlap from narrowband emission through the transition to laser-induced breakdown. Adapted from Ref. [148]

Experiments on TiO_2_ nanoparticles indicate that LII quickly transitions into a LIBS regime with increasing laser fluence. Zizak, De Iuliis, and co-workers have reported LII measurements on TiO_2_ nanoparticles in a series of publications [64, 146–149]. The measured emission spectra were found to consist of a broadband component resembling blackbody emission, superimposed with narrowband features [146]. These features were attributed to spontaneous relaxation of species that evaporate/sublime from the hot particle in an electronically excited state, or species that become excited as a consequence of gas phase processes (chemiluminescence) [153]. In many of these studies, the oxide nanoparticles were generated via a spray-flame process, which involve high flame temperatures (~ 2750–2950 K, as per Fig. 12) [148], in order to quickly evaporate the liquid precursor. This temperature is close to the peak particle temperatures (around 3150 K for a 50 ns delay and a fluence of 80 mJ/cm^2^), which makes particle sizing, derived from signal decay curves, difficult.

Excitation of TiO_2_ nanoparticles at 266 nm causes emission dominated by photoluminescence in the 400–600 nm range at low fluence (< 1 mJ/cm^2^), which rapidly decays and becomes undetectable within ~ 300 ns. Broadband emission was observed with fluences reported as 17 mJ/cm^2^, while higher fluence (56 mJ/cm^2^) caused strong overlap from narrowband emission through the transition to laser-induced breakdown (Fig. 12) [148]. At 355 nm excitation with a fluence of 350 mJ/cm^2^, Yi et al. [150] reported that laser-induced emission from TiO_2_ is initially dominated by narrowband emission but then relaxes within ~ 50 ns to a weak but mostly broadband LII-like signal. With 1064 nm excitation, in contrast, a broadband-dominated TiO_2_ signal can be observed with fluences up to 560 mJ/cm^2^, corresponding to particle temperatures between 500 and 1000 K higher than the equilibrium flame temperature, depending on the delay between laser peak position and detection gate [64]. The broadband signal generated with fluences above ~ 200 mJ/cm^2^ deviates from the expected Planck curve shape found at lower fluences. The authors attribute this to laser-induced defects that alter the optical properties of the particles [64], as well as nonuniform particle temperatures within the probe volume [149].

The small absorption cross-section of these nanoparticles makes their incandescence signal particularly susceptible to contamination from other emission, which may arise from interactions between the energetic UV photons, the nanoparticle, and species evaporated/sublimed from the nanoparticle [154]. Additional complications have been hypothesized as arising from microplasmas enveloping the nanoparticles, that may contaminate incandescence signal with bremsstrahlung radiation, even at low laser fluences [155, 156]. At sufficiently high laser fluences these microplasmas may consume the particle phase, giving rise to spectrally sharp atomic or ionic emissions indicative of the evaporated and ionized particle material [157]. This so-called phase-selective laser-induced breakdown (PS-LIBS) requires fluence levels beyond the LII plateau regime and its diagnostic capabilities for nanoparticle phase will be treated in some more detail in Sect. 7.1.

Murakami et al. [158] carried out measurements on 7 nm photocatalytic TiO_2_ powder using a pulsed UV laser. These authors report that the laser-heated nanoparticles emit OH-radicals, which interact with the laser pulse and emit fluorescence or photoluminescence.

**Fig. 13:**
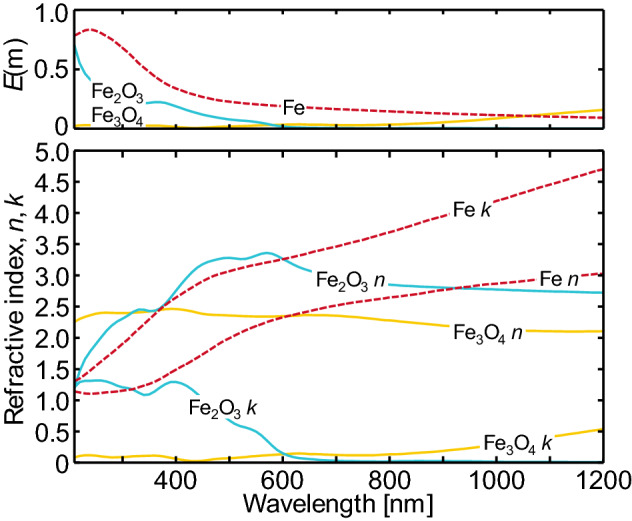
Refractive index and absorption function, *E*(**m**), as a function of wavelength for Fe_2_O_3_, Fe_3_O_4_, and solid Fe. Data from Ref. [115]

Many other oxides, especially brown- and black-colored transition-metal oxides, have strong interaction within the visible and near infrared light, such that particle heating is more easily accomplished. As an additional complication, in these cases, a variety of oxide phases exist and, from a thermodynamic point of view, less-oxidized phases are favored with increasing temperature (Fe_2_O_3_ → Fe_3_O_4_ → FeO → Fe) [159]. These phase transitions and chemical reduction mechanisms are endothermic and therefore should factor into the energy balance during laser heating and cooling. The optical properties of these phases are also distinct [115] (Fig. 13), so these transitions are likely to affect both the laser absorption cross-section and emission spectrum.

A further concern is that absorption-based laser-heating of nanoparticles may induce phase transitions and crystallite relaxation. Mendili et al. [160] reported an *γ*- to *α*-phase transition of Fe_2_O_3_ iron-oxide nanoparticles during continuous wave laser heating, as inferred from the background intensity and spectral widths of their corresponding Raman modes when excited with laser powers between 200 and 600 mW. Thermal accommodation coefficients between oxide nanoparticles and bath gases are also unknown and may change with particle phase, since the surface lattice and gas–surface potential well are both known to impact the TAC [135, 161]. It is, however, not clear how quickly these phase-transition and reduction processes occur relative to the timescales relevant to LII heating and cooling. Therefore, for oxide materials, the description of the energy and mass balance, as well as the optical properties leading to the measurable signal, is significantly more complex compared to elemental particles.

**Fig. 14:**
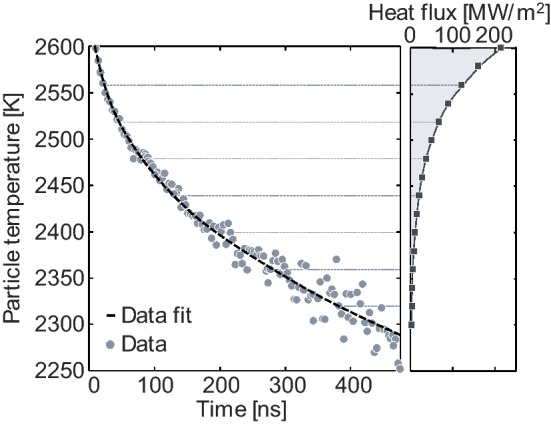
Time-dependent temperature of manganese oxide nanoparticles after laser heating and assessed heat flux. Quantification is determined from calibration of the LII data against particle size data from TEM. Adapted from [90]

In view of these complexities, Lehre et al. [90] take a pragmatic approach in their LII study of manganese oxide nanoparticles in which time-resolved LII traces are calibrated with additional TEM-inferred particle sizes using the thermal accommodation coefficient as a fitting constant (Fig. 14). Time-dependent temperatures are inferred by fitting a Planck distribution to recovered spectrum, with a peak temperature of 2800 K. The authors even derive particle size distributions from their measurements, which, considering the ill-posed nature of the problem and the uncertainty in the measurement model, is ambitious.

Dörr [162] adopted a similar technique to characterize the growth, aggregation, and volume concentration of Fe_2_O_3_ nanoparticles formed in low-pressure flame reactors using a combination of LII, extinction, and scattering measurements, and spectrally resolved chemiluminescence. Optical measurements were combined with particle mass spectrometry (PMS)-based particle sizing in the particle formation zone as an independent method. Additional reports have been published in brief conference proceedings on manganese oxide [151] and iron oxide [152], which, as already mentioned for TiO_2_ nanoparticles, show LII signals that decline very quickly in comparison to what is typical for carbonaceous nanoparticles.

While most LII studies on non-elemental nanoparticles have focused on oxides (Table 3), in an early work, Filippov et al. [89] carried out TiRe-LII measurements on aerosolized TiN nanoparticle powders. The authors observed faster LII signal intensity decays at higher laser fluences. A quantitative analysis of these curves using a standard LII measurement model seemed to indicate a bimodal size distribution: the first mode corresponds to particle aggregates with diameters in the range 20–120 nm, while the second mode peaks at much larger aggregate sizes of around 300 nm. Comparisons with TEM images taken from extracted samples prior to entering the cell also show primary particles of diameter around 10 nm, the presence of which is not indicated in the LII signal trace. This may either be a result of their small signal amplitude contribution due to low concentration and fast temporal signal decay, or—as the authors speculate—that these smaller particles are firmly bound to the larger aggregates (e.g., by sintering) forming compact units, where the signal is dominated by the slower cooling process of the larger entity. In view of the aforementioned complexities, considerable research is required in order to develop TiRe-LII into a reliable tool for quantitative investigations on oxide and nitride nanoparticles.

**Table 3:** LII studies on oxide and nitride nanoparticles

Material	Studies
Titania (TiO_2_)	Maffi et al. [146], Cignoli et al. [147], De Iuliis et al. [64, 148, 149], Yi et al. [150]
Iron oxides (Fe_2_O_3_, etc.)	Lehre et al. [90]*, Dörr et al. [151], Tribalet et al. [152]
Manganese oxide (MnO)	Lehre et al. [90]
Silica (SiO_2_)	Altman et al. [144]
Alumina (Al_2_O_3_)	Weeks and Duley [111]
Titanium nitride (TiN)	Filippov et al. [89]

*Lehre et al. [90] studied both iron and manganese oxide but only reported results for the latter

## Non-soot carbonaceous materials

**Fig. 15:**
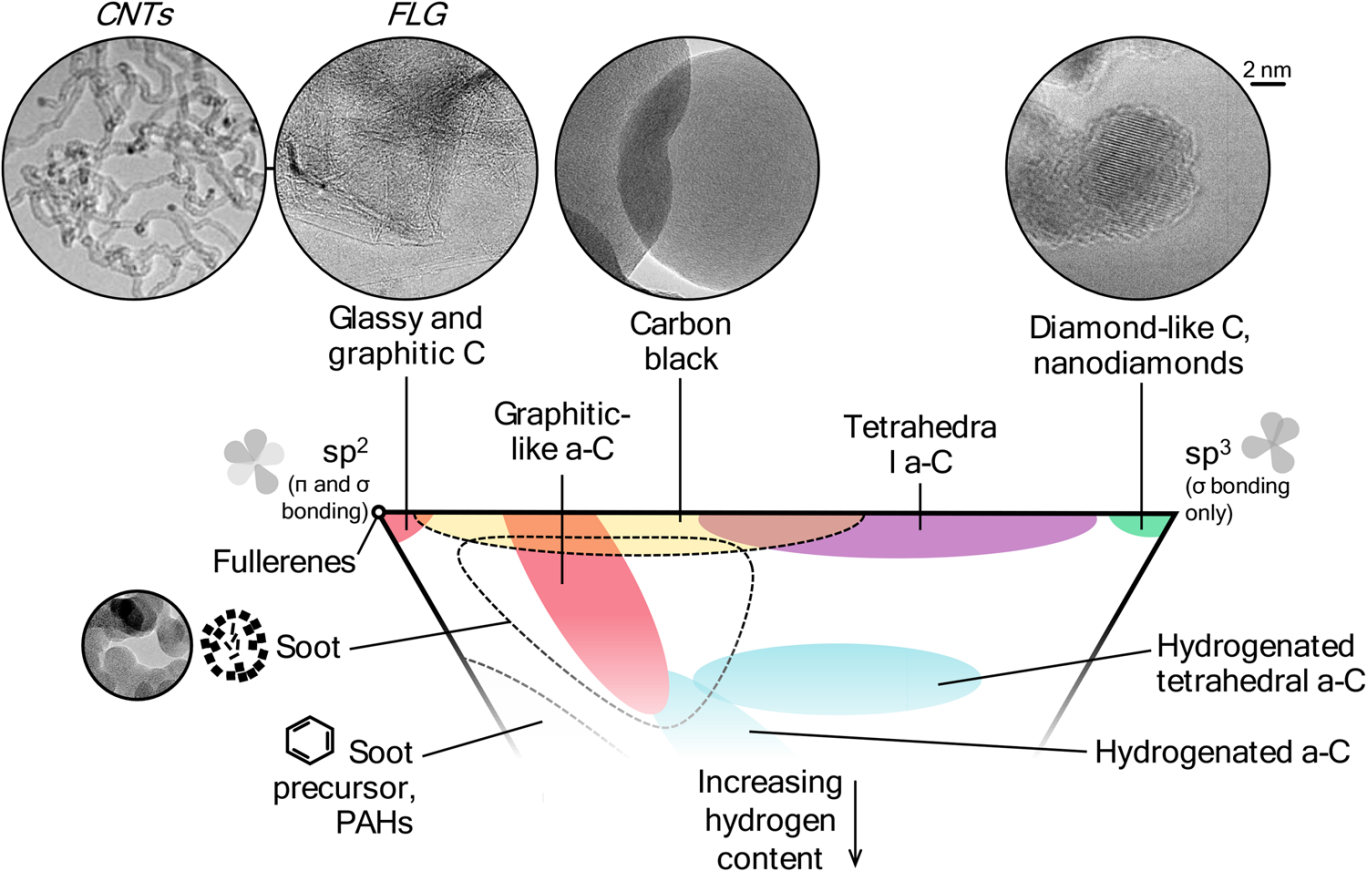
Schematics of carbon allotropes following from sp^2^ and sp^3^ hybridization and hydrogen content. “a-C” denotes amorphous carbon phases with short-range order only. Underlying ternary diagram adapted from Baldelli et al. [167], Ferrari & Robertson [168], and Russo et al. [169]. TEM images are taken from Cui et al. [170] for nanodiamond, from Vander Wal et al. [63] for soot and CNTs, and from Singh and Vander Wal [171] for carbon black

Engineered carbon nanomaterials are distinguished from soot based on their relatively low PAH and hydrogen content, as well as by how they are generated. Carbonaceous nanomaterials can be further classified by their internal structure, such as the relative prominence of sp^2^ versus sp^3^ hybridization and degree of long-range order. Figure 15 shows an overview of several carbon allotropes. LII has mostly been used to study the more-graphitic allotropes, including carbon nanotubes (CNTs), few-layer graphene (FLG) [41], most carbon blacks (which often also contain substantial amorphous domains), carbon nanodots [163], and carbon onions [164]. Carbon blacks are sufficiently similar to soot to be excluded from this review but were featured in the earliest LII study [111], where the authors aerosolized particles from a powder rather than observing a combustion process. It is also worth noting that carbon black is a potential reference material and is useful material for developing LII measurement models for soot, and particularly for elucidating how variations in internal structure, e.g., graphitization with soot aging, may affect the TiRe-LII signal [165, 166].

Carbon nanotubes (CNTs) are among the most frequently studied carbon allotropes due to their unique properties and industrial potency. Aerosol synthesis of CNTs is often done using metal catalyst nanoparticles. Puretzky et al. [172, 173] and Kokai et al. [174], for example, first measured LII from laser ablation-synthesized CNTs produced using a Co–Ni catalyst, noting the presence of blackbody emission from the C/Co/Ni plume as it progresses away from the wall. Vander Wal et al. [175, 176] studied TiRe-LII from flame-synthesized CNTs in the presence of Fe and Ni catalysts (the latter of which produced carbon nanofibers instead of CNTs), including double-pulse experiments and the first detailed, TiRe-LII decays for aerosolized CNTs. Cau et al. [177] characterized CNTs formed through laser ablation synthesis in the presence of iron, cobalt, nickel, and yttrium catalysts and soot. Changes in the incandescence signal were connected to particle growth; a sharp increase in the incandescence signal aligned with the addition of the metal catalysts, suggesting extra emission from the CNTs and the catalysts. The relative contributions of the CNTs and the metal catalyst nanoparticles remains an open question across these studies. Mitrani and coworkers [178, 179] carried out LII on multi-walled CNTs (MWCNTs) synthesized in a carbon arc reactor, avoiding issues with the presence of metallic nanoparticles, and explored electron emission from the laser-heated particles. Vivien et al. [180] conducted TiRe-LII on CNT colloidal suspensions in chloroform and water. Due to the surrounding liquid, time-resolved emission signals were exceptionally short, with exponential decay times on the order of 10 ns. Zeng et al. [181] and Lim et al. [182, 183] performed LII measurements on CNT films, recording emission spectra and measurable incandescence at lower fluences. Overall, these studies demonstrate that laser-heated CNTs produce measurable signals, but have not definitively shown that the signals can be distinguished from those of the background soot or catalyst materials that may also be present, and, for the most part, they have not been interpreted in a quantitative context.

Other allotropes feature less commonly in the literature. Carbon arc reactors have been used to generate a range of nanomaterials, including CNTs, fullerenes, carbon nanowires, and nanosheets; many of these structures have been probed with TiRe-LII [178, 179, 184–186]. Yatom et al. [186] used LII imaging to map the spatial distribution of particles exiting an arc discharge, for comparison against a computational fluid dynamics model. Musikhin et al. [41] carried out LII on FLG particles formed within a microwave plasma reactor. LII signal intensity was correlated with FLG volume fraction estimates from other diagnostics. This data was compared with measurements on soot particles generated using different precursors. Long et al. [163] carried out LII on individual carbonaceous particles (boron-doped nanodiamonds, featuring sp^3^ hybridization; carbon dots; and graphene platelets) trapped using a single nanoparticle mass spectrometer. The nanoparticles were heated with 532 or 1064 nm pulses. The recovered spectra were compared to elucidate the connection between carbon structure and the spectral emission cross-section (expressed as a spectral emissivity, although this bulk property is inappropriate for nanoparticles). Measurements on pure nanodiamonds were not possible due to their low absorption cross-section at the laser wavelength.

In terms of emission spectra, laser-heated carbonaceous nanoparticles are largely consistent across the alloptropes; this is likely due to the fact that most of these particles emit in the Rayleigh limit of Mie theory, so the constituent dipoles oscillate in phase (i.e., no phase shift) and the absorption cross-section only depends on dipole number density and not particle morphology. Osswald et al. [164] measured emission spectra through the entire visible range for carbon onions, carbon black, MWCNTs, and nanodiamond, following excitation at 325 nm. While the study focused on Raman analysis, the thermal emission curves were again similar, with periodic differences between the curves attributed to measurement uncertainties. Musikhin et al. [41] used a streak camera to measure the emission spectra of laser-heated FLG particles over time, with emission showing a similar response through the visible spectrum. Similar observations were also made for CNTs by Vander Wal et al. [175] and Zeng et al. [181]. Kokai et al. [174] made spectral measurements of CNTs and identified spectra for the metal catalyst species and presumed bremsstrahlung emission. Nevertheless, the emission spectrum may be affected by laser-induced changes in atomic structure, e.g., graphitization. For example, Long et al. [163] showed that the emission spectra from graphite evolves differently with laser heating compared to boron-doped nanodiamond with increasing laser fluences.

**Fig. 16:**
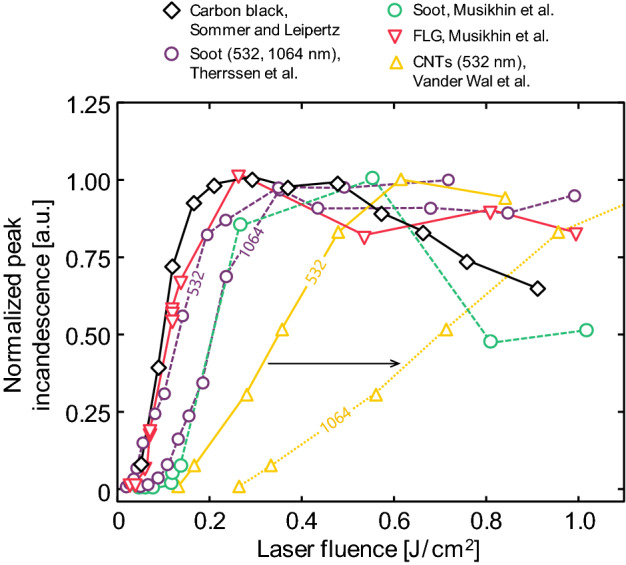
Normalized peak incandescence fluence curves for three allotropes of carbon: carbon black [187], soot [41, 189], few-layer graphene (FLG) [41], and carbon nanotubes (CNTs) [175]. Results are for excitation at 1064 nm, unless otherwise stated. The 1064 nm curve for the CNTs (yellow, dotted line) is a predicted curve, assuming the Rayleigh approximation (which has been shown to be valid for a range of carbon allotropes, e.g., [52]) and a constant *E*(**m**) (which is roughly true, ± 15% [189, 190]). Dashed lines correspond to soot. Carbon black results are for a re-aerosolized colloid. CNTs were characterized immediately following synthesis by pyrolysis. FLG and plasma soot (Musikhin et al. [41]) were characterized following synthesis in a microwave plasma reactor, while flame soot (Therssen et al. [189]) was characterized above a McKenna burner. See Sipkens et al. [21] for further discussion of fluence curve regimes and their variability, as well as a method to non-dimensionalize fluence curves

The variation of peak temperature or peak incandescence with laser fluence (a so-called “fluence curve”) also provides important insight into absorption efficiency and evaporation. These measurements have been carried out for carbon black [187, 188], CNTs [175, 180, 181], and FLG [41] (Fig. 16). All these studies show the same trend: The peak signal increases linearly with fluence until the peak temperature approaches the sublimation temperature, after which both the incandescence and temperature plateau or, in the case of incandescence, eventually declines as the fluence increases due to sublimation mass loss. Rulik et al. [188] constructed a fluence curve from LII measurements on carbon black particles in a colloid. Musikhin et al. [41] compared the fluence curve for FLG against soot, with FLG exhibiting a faster rise in the low-fluence regime, a slightly higher plateau temperature, and the peak incandescence potentially suggesting less sublimation. Overall, the normalized intensity for the CNTs [175] increases much more slowly than the other carbon allotropes.

The morphology of CNTs and FLG particles differ substantially from the particle aggregates typical of soot and carbon black. Nevertheless, TiRe-LII shows promise for interrogating this morphology, since signal decay rate is roughly proportional to the heat transfer surface area. Cau et al. [177] interpreted TiRe-LII signals from soot and CNTs using a model developed by Krüger et al. [191] for soot, but without accounting for the morphological differences between these two material types. Mitrani and Shneider [179] presented a simple heat transfer model for MWCNTs, which accounted for their cylindrical shape and allowed for non-uniform temperatures within the nanomaterial. Musikhin et al. [41] quantitatively interpreted TiRe-LII data from FLG particles using an effective diameter to account for the larger surface area (estimated to be about double that of soot, based on Brunner-Emmett-Teller analysis). Temperature decays were more rapid for FLG compared to soot, likely due to a combination of the higher surface area-to-volume ratio of FLG relative to soot and the higher peak temperature predicted to be reached by FLG. At present, since most of the studied allotropes feature sp^2^ hybridization, the heat transfer models used for these other materials are the same as those applied to soot. However, there are likely differences in some of the properties, most notably with respect to the evaporation submodel [41], that require further study and refinement to improve accuracy.

## Complex and composite nanomaterials

Applications of LII to more complex or composite nanomaterials are relatively rare. Candidate nanomaterials include homogeneous mixtures of two or more compounds, core–shell arrangements, or special morphologies, such as prisms or hollow spheres. To date, LII studies have only considered carbon-coated iron in a series of papers by Eremin and coworkers [49–51] and carbon-coated titania [192]. A major obstacle to implementing LII on these materials are their poorly characterized properties, in particular in contrast to elemental particles. Approximate optical and thermophysical properties are often computed via “mixing rules” from known values of the pure constituents making up the materials system, weighted by their respective mass or volume fraction in the final product, although the suitability of these approaches may, in some cases, be questionable. An alternative approach is to use TiRe-LII to investigate the very thermophysical and optical properties that prevent current quantitative analyses by supplementing TiRe-LII measurements with information from other techniques (e.g., TEM).

Eremin et al. [49] generated nanoparticles by laser-photolysis of iron pentacarbonyl at 266 nm; pure iron nanoparticles were generated using FeCO_5_/Ar mixtures before either methane or acetylene were added to the mixture as a means to coat the iron nanoparticles with a thin carbon layer. Particle growth was tracked via time-resolved optical extinction. The acetylene-containing mixture produced carbon-coated nanoparticles, attributed to the presence of catalytically active surface sites, while the mixture containing methane did not.

**Fig. 17:**
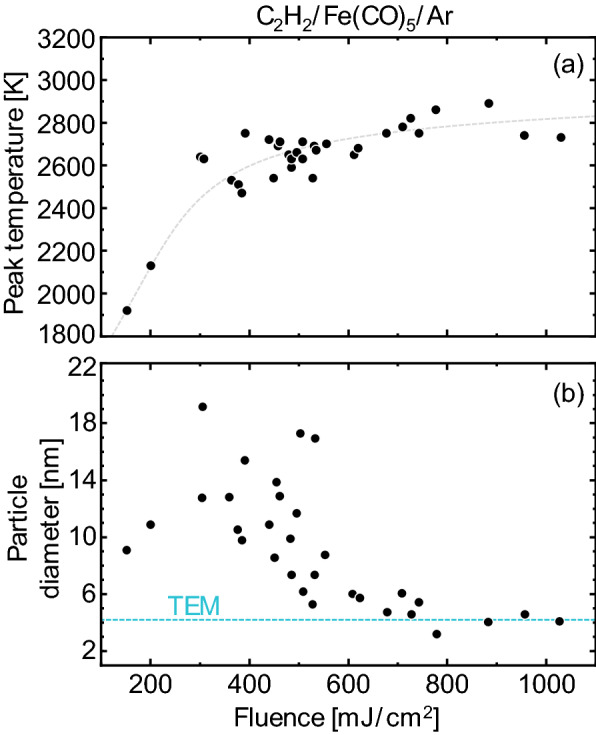
Peak temperature (**a**) and mean particle diameter (**b**) of laser-heated particles obtained in a mixture of 0.5 bar C_2_H_2_ + 20 mbar Fe(CO)_5_ + 0.5 bar Ar in dependence of laser fluence. In **a**, the faint dashed line is only intended to guide the eye. In **b**, the horizontal dashed line represents the particle size retrieved from particle sampling and TEM image analysis. Adapted from Figs. 10 and 12 in Ref. [50]

The same group extended the photolytically initiated iron–carbon nanoparticle synthesis to consider additional argon-diluted mixtures of Fe(CO)_5_ with benzene, toluene, or butanol [50]. The mean particle size was retrieved by pulsed two-color TiRe-LII with a 1064 nm heating laser. No information is provided about the spectroscopic model, thus, based on previous work by this group, it is assumed that they adopted Eq. (10) with an *E*(**m**) ratio of unity. This may be questionable given that the refractive index of iron varies over the detection wavelength and the Rayleigh model is inapplicable, cf. Sec. 2.1. Peak pyrometric temperatures of laser-heated, carbon-encapsulated iron particles increased from 1900 to 2850 K when the laser fluence increased from 150 to 900 mJ/cm^2^ (Fig. 17a), which is much lower than soot particles of similar size but higher than for pure iron nanoparticles (around 2400 K). Particle sizing was achieved using a heat transfer model employing a linear approximation between solid and liquid for density, and heat capacity of bulk iron, a thermal accommodation coefficient of 0.44, and the vaporization properties of graphite (as vaporized species will only issue from the shell). The presence of the coating undoubtedly complicates the evaporation process: since the electromagnetic field of the laser heats both the carbon layer and (especially) the metal core, there must be a mechanism through which iron vapor from the core escapes through the carbon layer, which may lead to fragmentation of the nanoparticle into smaller agglomerate structures (Fig. 17b). The authors explain the discrepancies between both size measurement methods by, among other issues, uncertainties/deficiencies in the LII model.

**Fig. 18:**
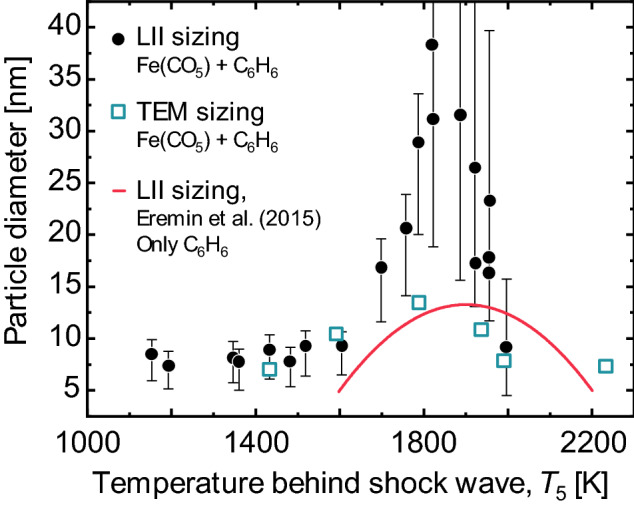
Example of application of LII to measure the diameter of carbon-encapsulated iron particles as a function of the temperature behind the shock wave, *T*_5_. Adapted from [51]. Data for 1% C_6_H_6_ in Ar is from an earlier work [193]

In a follow-up work from the same group [51], carbon-encapsulated iron particles were synthesized by shock-heating mixtures of Fe(CO)_5_, Ar, and either C_2_H_2_ or C_6_H_6_. TiRe-LII was triggered after the reflected shock wave passed the probe volume and allowed for in situ determination of particle size, with laser fluences kept between 70 and 150 mJ/cm^2^ to avoid particle evaporation and emission from evaporating atomic/molecular species, while providing a reasonable signal-to-noise ratio. The generated nanoparticles typically feature an iron core of 3–10 nm covered by a carbon shell containing between 2 and 20 atomic layers, with benzene producing the smaller particles. In the LII model refined from their previous work, the mass ratio between the iron core and carbon shell was estimated from TEM images and used to calculate a mean density. The authors found that when utilizing thermal accommodation coefficients between 0.35 and 0.46 reported in the literature for similar systems, model-retrieved particle sizes deviated between 21 and 25% from the values with *α* = 0.44 in experiments in mixtures with benzene as soot precursor. A thorough error analysis led the authors to claim uncertainty ranges in LII particle sizing for an iron:carbon mass ratio of 5:1 between – 31 and + 17%, and that density, heat capacity, and TAC are the most sensitive parameters affecting deduced particle sizes. Unfortunately, predicted mean primary particle sizes deviated from TEM results (Fig. 18); slower cooling of the aggregate structure may be misinterpreted as larger particles, and inferred sizes showed an unexpected dependence on the temperature behind the shock wave.

Ren et al. [192] investigated carbon-coated TiO_2_ nanoparticles formed initially in the oxidation zone of a counterflow flame with ethylene as a fuel entering the interaction zone from above and titanium iso-propoxide (TTIP, Ti(C_3_H_7_O)_4_, loading rate typically 100 μL/min) as the prevaporized precursor (typically around 1400 ppm) mixed in the oxygen flow from below; both flows were diluted with N_2_. In such a configuration, the metal oxide nanoparticles quickly grow on the oxidizer side of the flame sheet without interaction with the carbon-containing fuel, whereas their carbonaceous shell is formed as the nanoparticles travel through the fuel side of the flame. Specific crystalline structures and coating thicknesses may be realized by controlling particle temperature–time histories as they transition through flame regions of varying temperature and gaseous composition.

**Fig. 19:**
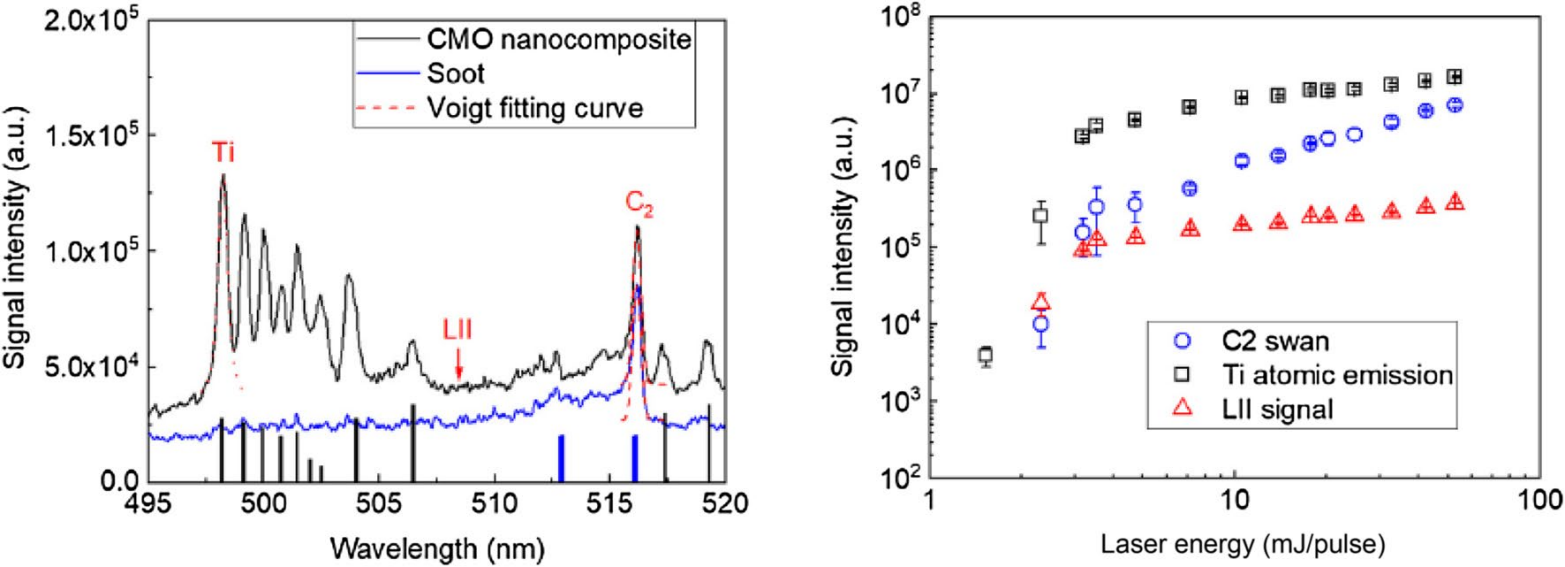
Spectroscopy of carbon–metal oxide (CMO) nanocomposites (black line) and the pure soot nanoparticles (blue line), as well as the Voigt fitting curves (red dotted lines). The laser beam energy is 18.9 mJ/pulse and it crosses the axial position of 6.93 mm (from the fuel nozzle). Vertical lines mark spectral position and line strength of gas phase optical transitions of Ti (black) and C (blue). From Ref. [192]

LII was carried out with a 532 nm focused laser pulse. Emission was detected using a spectrometer coupled to an ICCD camera. Measurements allowed for simultaneous detection of C_2_^*^ Swan emission (at 516.1 nm); for high enough laser fluences, Ti^*^ atomic emission (the 498.17 nm line, see discussion of PS-LIBS in Sect. 7); and, around 508.6 nm, a continuous spectrum, attributed to incandescence. The Ti^*^ atomic emission was used to distinguish zones of soot from those containing carbon–metal oxide (CMO) particles (Fig. 19). The LII and spectral information allowed for spatially resolved measurement of particle concentration and elemental composition. However, as the authors state: “the LII signal is also a function of the particle composition due to the varying complex refractive index, density, capacity, etc. Therefore, it is challenging to correlate the LII signal with the particle volume fraction quantitatively.” The authors complemented these results with TEM (size) and X-ray diffraction (XRD) (chemical analysis).

From the laser fluence dependence of the LII, PS-LIBS, and the C_2_^*^ Swan band emission intensities (right graph in Fig. 19), as well as TEM image analysis, the authors concluded that the saturated LII signal (i.e., for laser fluences in the plateau regime) can serve as an indicator for the total particle volume fraction, while the Ti atomic and C_2_ molecular emissions are a measure for the elemental (metal vs. carbon) composition of the CMO nanocomposite. This data, along with the intensity profiles along the burner axial direction, was used to derive a CMO formation mechanism, with particle size (i.e., TiO_2_ core) increasing with precursor seeding rate keeping an almost constant carbonaceous shell thickness. Contrary to the laser photolysis [50] or shock-tube pyrolysis [51] initiated synthesis processes of carbon-coated iron-oxide by Eremin et al., the counterflow flame geometry allows for truly sequential coating of the respective metal-oxide cores.

These largely empirical results highlight the promise of TiRe-LII as a tool for characterizing composite and otherwise complex nanoparticles. As it stands, qualitative results remain useful in assessing changes in nanoparticle characteristics and determining the location where the changes happen in a reaction system and thus provide a route to further study these unique nanomaterials. However, considerable further research is needed to develop the sophisticated spectroscopic and heat transfer models needed to support quantitative analysis of these materials.

## Combined and complementary diagnostics

### Other sources of laser-induced emission

The laser pulse used to induce an incandescence signal may also elicit other types of signals from the nanoparticles, collectively referred to as non-incandescent laser-induced emission (LIE). While these signals are often a nuisance, particularly when they are difficult to distinguish from incandescence, they may also provide additional insight about the nanoparticle quantities-of-interest that complement the information contained in the LII signal.

Most non-incandescent LIE arises from species evaporated from the nanoparticle by the LII excitation pulse. For example, as noted in Sect. 3, a portion of the prompt signal from laser-heated metal nanoparticles may arise from bremsstrahlung between thermionically induced emission (evaporation) of electrons and neutral bath gas atoms [138]. Because bremsstrahlung emission has a broadband character that closely resembles incandescence from the nanoparticle, this type of LIE may significantly bias quantities inferred from the LII signal, particularly at measurement times close to the peak incandescence.

**Fig. 20:**
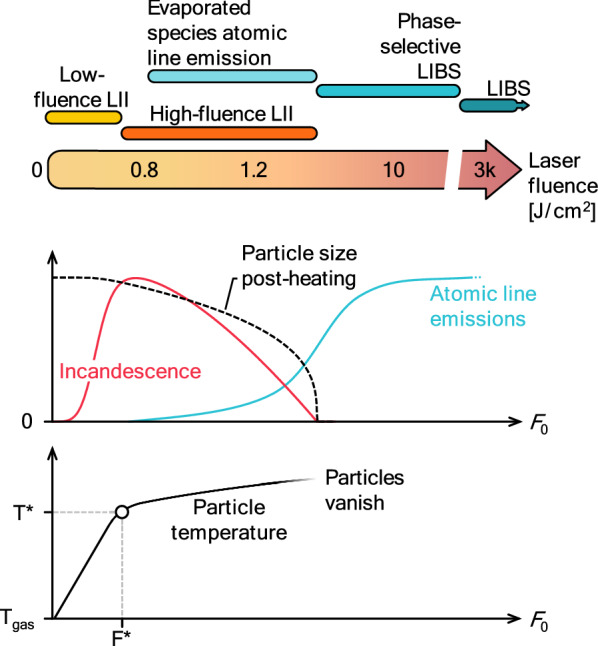
Schematic demonstrating the typical progression of the LII and LIBS signals as the laser fluence is increased for an excitation wavelength of 1064 nm, adapted from Ref. [196]. Values of laser fluence for this progression will vary substantially between materials and experimental conditions, including the gas temperature and excitation wavelengths

Atomic emission lines, such as those observed in Fig. 19 for carbon-coated titania or emission from the C_2_ swan bands for carbonaceous materials, are another common form of laser-induced emission. The underlying mechanism depends on the fluence regime, shown schematically in Fig. 20. At low fluences, atomic line emission is not observed. As the fluence is increased, atoms and molecules eventually evaporate from the nanoparticle. Since the phase interface is at local thermal equilibrium, as discussed in Sect. 2.2, the molecules evaporate with translational and internal energies that obey a Boltzmann distribution at the particle temperature. A known portion of the evaporated atoms and molecules will therefore be electronically excited, and will spontaneously relax in the gas state according to the Einstein coefficient for a given transition. Menser et al. [92, 194] exploited this phenomenon to investigate the evaporative behavior of liquid silicon and liquid germanium nanoparticles. This was done by systematically varying the laser fluence, and measuring the corresponding peak line intensity of key transitions (e.g., 3s^2^ 3p^4^ s ^1^*P*° 1 → 3s^2^ 3p^2 1^*D* 2 at 288 nm for Si [195]) using a streak camera.

**Fig. 21:**
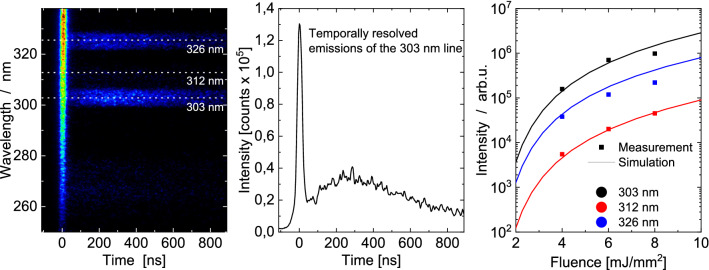
(left) Streak camera image of a PS-LIBS measurement of germanium [197] nanoparticles in a microwave plasma synthesis reactor using a laser fluence of 1,400 mJ/cm^2^ with a pulse length of approximately 8 ns at an excitation wavelength of 1064 nm. (center) Temporal evolution of the 303-nm line. (right) Integrated intensity of the prompt peak for the three indicated lines. The dots show the measurements and the solid lines give the solution of the atomic line emission model

Figure 21 shows how the peak line strength varies as a function of laser energy. These measurements constitute, to the best of the authors’ knowledge, the first independent verification of the LII evaporation model, Eqs. (13)–(15). Moreover, Menser et al. [92] combined the peak atomic emission intensity versus fluence, peak nanoparticle temperature versus fluence, and individual temperature decay traces to develop probabilistic estimates of the Antoine parameters. Intriguingly, the detected atomic emission signal intensities showed two distinct modes: a short peak temporally aligned with the heating pulse, which corresponds to the evaporation mechanism described previously, and a second, longer lasting peak having a characteristic relaxation time on the order of microseconds. The second peak suggests that some gas-phase process must be producing electronically excited silicon and germanium atoms, which then spontaneously relax and emit radiation. The authors speculate that electron impact excitation is a likely source, and that the energetic electrons originate from a microplasma heated through inverse bremsstrahlung.

At still higher fluences, Zhang et al. [198] showed the existence of a regime in which the nanoparticles are converted into a plasma, without affecting the surrounding gas. The beginning of this measurement regime—corresponding to phase-selective laser-induced breakdown spectroscopy (PS-LIBS)—marks the upper fluence limit for LII. At the same time, these fluences are well below what is theoretically needed for breakdown of the gas phase in conventional LIBS measurements. Tse and coworkers [22, 145, 198] show the spectrally resolved emission of the laser-generated plasma during the synthesis process of titania nanoparticles. Strong line emissions between 497 and 505 nm were associated to atomic titanium (Ti I, cf. Fig. 19), while a line at 376 nm originates from titanium ions (Ti II), but no emission from the bath gas species was observed. Stodt et al. [199] present a similar approach, but for iron oxide (Fe_x_O_y_) nanoparticles formed in a spray flame, where strong line emissions between 404 and 407 nm originate from atomic iron (Fe I). Guo et al. [200] applied PS-LIBS to monitor two-dimensional nanoparticle distributions in synthesis flames by using a 532 nm laser light sheet. They observed various lines of vanadium (V I) with the strongest ones between 438 and 441 nm. The lines become observable temporally close to the laser pulse and last for approximately 20 ns, which is consistent with the expected fluorescence lifetime between 8 and 17 ns.

While the most obvious application of PS-LIBS is to determine the chemical composition of the nanoparticle phase from the relative strengths of atomic emission lines, the fluence level of PS-LIBS saturation can also be used to estimate the total nanoparticle mass/volume concentration in the observation volume, which can complement LII measurements. Zhang et al. [22, 198] and Carranza et al. [201] showed that the measured atomic emission intensity correlates with the average particle diameter in the observation volume. While Zhang et al. scan the fluences until the signal saturates, Carranza et al. vary the particle diameter with a constant fluence and determined a fixed fluence limits the largest particle size for which the plasma energy is not sufficiently high to evaporate the particle fully. The fluence where phase-selectivity ends corresponds to the gas breakdown threshold, above which the gas phase transitions into a plasma [202, 203].

If the particles have suitable chromophores, several authors have shown that conclusions about the particle size can also be drawn from the photoluminescence emitted after laser excitation [18, 204].

### Complementary techniques

Several techniques have been used in conjunction with TiRe-LII, as summarized in Table 4. Line-of-sight attenuation (LOSA), for example, enables a route to measure extinction coefficients and, by extension, the spectral absorption cross-sections needed for the LII spectroscopic submodel (see Sect. 2.1). Within this context, one would typically use a broadband light source and spectrometer–camera combination [197, 205] to obtain spectral information over both the LII absorption and detection wavelengths. LOSA measurements targeted the optical properties of several TiRe-LII materials systems, including aerosols of liquid germanium [206], silicon [40, 95], and copper [40] nanoparticles. Depending on the complexity of the underlying materials system, LOSA can be used to infer more fundamental information that can be used to parameterize physical models, such as the Drude model for metals, the Lorentz oscillator model for materials with interband transitions, or semiconductors.

**Table 4:** Complementary diagnostics used in conjunction with TiRe-LII on non-carbonaceous nanoparticles and FLG

Diagnostic	Complementary properties	Sample studies
Atomic line emission spectroscopy	Evaporation model parameters	Menser et al. [92]
Line-of-sight attenuation (LOSA)	Absorption function	Menser et al. [46], Daun et al. [40], Asif et al. [95]
Raman scattering	Particle size, crystal structure	Liu et al. [17]
Elastic light scattering	Radius of gyration, aggregation	Huber and Will [207]
Dynamic light scattering (DLS)	Particle size	Sipkens et al. [60, 61]
Electron microscopy (TEM, SEM, HR-TEM)	Primary particle diameter, aggregation, internal structure	Vander Wal et al. [42], Starke et al. [91, 147], Kock et al. [58], Lehre et al. [90], Eremin et al. [59, 85, 97], Cignoli et al. [147], Sipkens et al. [60, 72], Menser et al
Brunauer–Emmett–Teller (BET) analysis	Particle surface area	Sipkens et al. [72]

Sample studies are those studies in which the diagnostic was used directly for or with the intention of knowledge transfer to future TiRe-LII experiments

At laser fluences below the threshold that produces detectable incandescence, spontaneous Raman scattering has been successfully applied to gas-borne TiO_2_ nanoparticles in a flame reactor for in situ identification of the crystal structure [17]. The dependence of the Raman signal on particle size was exploited by Meier et al. [208] in order to infer information about the diameter of silicon nanoparticles extracted from a microwave plasma reactor.

Elastic light scattering has been carried out using either pulsed or continuous-wave lasers, sometimes in parallel with LII studies. In the case of spherical particles that satisfy the Rayleigh criterion (*x*_p_ << 1, ||**m*** · x*_p_|| << 1), the scattering cross-section, *C*_sca,λ_, scales with *d*_p_^6^ and, thus, strongly depends on particle size and wavelength. Santra et al. [209] used Rayleigh scattering intensities from two laser wavelengths (λ_1_ = 1064 nm and λ_2_ = 532 nm in horizontal and vertical polarization, respectively) to measure the aspect ratio length over diameter of carbon nanotubes.

The information content of the light scattering data can be enhanced by measuring the scattered light at multiple wavelengths or multiple angles; these two dimensions influence the scattering measurement model via the modulus of the scattering wavevector, *q* = 4πλ^–1^sin(*θ*), where *θ* is the angle between the direction of laser propagation and the detection view angle [53, 210]. Angular resolution may be obtained by using multiple sensors, or, in the case of a steady target, mounting a sensor on a goniometer (e.g., Link et al. [211]). Martins et al. [212] present a 2D multi-angle light scattering technique using six CCD cameras that are positioned on a circle between 10° and 90° in 16° steps around the burner to image the scattered light of a frequency-doubled Nd:YAG laser. In wide-angle light scattering (WALS), a parabolic mirror is used to image a continuous range of scattering angles. Huber and coworkers detected scattering between 10° and 170° to determine mean particle diameters and particle size distributions for soot [213], silica [207] and ethanol droplets [214].

Often LII and elastic light scattering may be combined to improve the robustness of the recovered variables. Combined elastic scattering and TiRe-LII measurements are particularly appealing in the case of aggregated nanoparticles, since the scattering measurement is more sensitive to the aggregate structure, while the TiRe-LII data indicates the primary particle size. As such, using light scattering in combination with laser-induced incandescence enables the detection of the radius of gyration of soot particle aggregates [215]. While this simultaneous approach is quite common for measuring soot aggregates (e.g., Refs. [216–218]), it has not yet been used to analyze engineered nanoparticle data and present an area of future study.

While it lies outside the scope of this review, it is worth noting that the SP2 instrument [219], which uses a continuous wave laser to irradiate nanoparticles and then measures the incandescence and elastic light scattering signals, has been used to determine the size and composition of tungsten, silicon, and graphite particles, as well as soot.

In many works reported in literature, TiRe-LII measurements are calibrated against ex situ techniques like transmission electron microscopy (TEM), particle mass spectrometry (PMS), scanning mobility particle sizing (SMPS), and Brunauer–Emmett–Teller (BET) analysis. Each of these measurement methods can determine specific nanoparticle properties, but they have different interpretations in the context of TiRe-LII-inferred sizes. In principle, both TEM and TiRe-LII may be used to infer primary particle size, although, in the case of TiRe-LII, the particle diameter corresponds to the specific heat transfer area in the same way that SMPS measurements correspond to a “mobility diameter”. While TEM measurements are widely considered the “gold standard” for particle sizing, especially in the context of the many LII measurement model uncertainties addressed in this paper, it is important to acknowledge that TEM-derived particle sizes may differ to those found in the gas phase. Reasons for this include: inadequate sample sizes due to the laborious nature of TEM analysis (even with automation); artifacts introduced by the extraction process (e.g., non-isokinetic sampling, aggregation in the sampling line); and surface forces [220] that distort non-rigid particles and agglomerates on the TEM grid. The BET technique provides the total surface in units of m^2^/g through surface desorption of a gas (most often nitrogen). The BET-inferred specific surface area is influenced by agglomerate structures of the product powder and the value can be strongly affected in case a second (large) particle phase is present. Pabisch et al. [221] showed that the particle sizes measured by BET underestimate the mean particle diameter compared to TEM. A scanning mobility particle sizer (SMPS) can be used either to preselect particles for a systematic analysis of predefined nanoparticle sizes or to measure the particle size classes of synthesized nanoparticles downstream from a reactor. A SMPS determines the mobility/hydrodynamic diameter, which for agglomerates or aggregates deviates significantly from the primary diameter assessed by LII. The mobility diameter is strongly influenced by the morphology of the particle powder and the particle shape. As a consequence, the determined particle sizes may be overestimated by 10–20% compared to TEM [222]. Overall, comparing particle sizes across various methods is not straightforward and requires an understanding of how the respective measurement principle interacts with a specific material.

## Current challenges and outlook

While laser-induced incandescence is a standard combustion diagnostic for measuring soot primary particle size and volume fraction, it is increasingly applied to characterize other types of aerosols, including metal, metal oxide, and manufactured carbonaceous particles. This trend is driven, in large part, by the increasing industrial relevance of nanoparticles, which are often produced in the gas phase.

Although the same basic LII measurement principle applies to both soot and synthetic nanoparticles, there are notable differences between these two scenarios. In the case of aerosols of elemental nanoparticles, the thermophysical properties needed for the LII measurement model are often much better known compared to those of soot, which vary with fuel composition and local combustion conditions, and evolve as soot “ages”. Moreover, many synthetic aerosols consist of “pure” spherical nanoparticles that obey a narrow size distribution, while soot particles usually have a more complicated and heterogeneous aggregate structure, and both primary particle and aggregate sizes often have wide size distributions. For these reasons, LII measurements on well-characterized nanoparticle systems may be used to better understand the fundamental aspects of electromagnetic theory and transport phenomena that underlie this diagnostic. These lessons can then be applied to more complex measurement scenarios, including soot. LII may also be deployed to carry out more fundamental scientific inquiries, e.g., to determine the thermophysical properties of materials at extreme temperatures and thermal accommodation coefficients between various gas/surface systems, both of which are difficult to infer from experiments on larger-scale materials.

On the other hand, in the case of more complex nanoparticles (e.g., those having heterogeneous structure and composition), LII model parameters may be difficult or impossible to quantify. In these scenarios the measurement model may need to be purely empirical, derived by fitting simulated LII traces with measurements in the context of nanoparticle sizes obtained through non-LII means (e.g., TEM analysis of extracted nanopowder). A particular challenge concerns the spectroscopic models that connect the measured spectral intensity to the nanoparticle temperature. While the spectral absorption cross-sections of soot particles can almost always be modeled using the Rayleigh approximation, this is not the case for synthetic nanoparticles, particularly those made of metal. Modeling the radiative properties of spherical metal nanoparticles requires the full Mie equations, which depend on the particle diameter. For non-spherical nanoparticles, even more sophisticated techniques, like the discrete dipole approximation, are needed to quantify the spectral absorption cross-section; otherwise, an empirical “fitting” approach would again need to be employed.

A further complication concerns non-incandescent laser-induced emission (LIE), which, under some experimental conditions, may contaminate LII signals and complicate data analysis. On the other hand, non-incandescent LIE may provide additional information about the aerosol. For example, at higher fluences, atoms and molecules evaporated from the nanoparticle produce atomic emission lines that can be used to determine nanoparticle composition, and, in some cases size, volume fraction, and other properties.

The capabilities of LII will continue to evolve in lockstep with the number and range of applications for synthetic nanoparticles. This diagnostic will prove particularly important for designing and controlling gas phase synthesis routes used to produce complex nanostructures, including few-layer graphene particles “decorated” with silicon nanoparticles, aggregates of metal and semiconductor nanoparticles, metal alloys, and core–shell structures. Analyzing these nanoparticle types will require a more detailed understanding of the spectroscopic and transport processes that connect the LII signal to the aerosol quantities-of-interest than is presently available. However, higher-fidelity LII measurement models are being developed using sophisticated electromagnetic and molecular dynamics simulations, enabled by high performance computing and machine learning. Parallel advancements in optoelectronics hardware (e.g., low-cost gated PMTs, picosecond pulsed lasers, and streak cameras) are presently being exploited to develop highly sensitive LII systems that are far more informative compared to traditional single-pulse, two-wavelength detection setups. In this context, particularly potent diagnostics may be realized by combining LII with complementary optical techniques, e.g., elastic and inelastic scattering and plasma signals related to laser-induced breakdown spectroscopy. Statistical analysis techniques must also be developed in order to fully exploit advancements in theoretical modeling and hardware. Bayesian-based approaches are particularly useful for quantifying the uncertainty in LII-derived quantities, synthesizing data from multiple diagnostics, and even developing LII measurement models.

The diagnostic tools enabled through these advancements will allow engineers to develop new types of synthetic nanoparticles and applications, allow combustion researchers to understand soot formation and its impact on human health and the environment, and equip scientists who wish to advance the frontiers of knowledge of thermodynamics, electromagnetics, and transport phenomena at the nanoscale.
